# Complete Mitochondrial Genomes of Five Racerunners (Lacertidae: *Eremias*) and Comparison with Other Lacertids: Insights into the Structure and Evolution of the Control Region

**DOI:** 10.3390/genes13050726

**Published:** 2022-04-21

**Authors:** Lili Tian, Xianguang Guo

**Affiliations:** 1 Chengdu Institute of Biology, Chinese Academy of Sciences, Chengdu 610041, China; tianlili21@mails.ucas.ac.cn; 2 University of Chinese Academy of Sciences, Beijing 100049, China

**Keywords:** mitochondrial genome, next-generation sequencing, Lacertidae, secondary structure, control region, evolution, phylogeny

## Abstract

Comparative studies on mitochondrial genomes (mitogenomes) as well as the structure and evolution of the mitochondrial control region are few in the Lacertidae family. Here, the complete mitogenomes of five individuals of *Eremias scripta* (2 individuals), *Eremias nikolskii*, *Eremias szczerbaki*, and *Eremias yarkandensis* were determined using next-generation sequencing and were compared with other lacertids available in GenBank. The circular mitogenomes comprised the standard set of 13 protein-coding genes (PCGs), 22 transfer RNA genes, 2 ribosomal RNA genes and a long non-coding control region (CR). The extent of purifying selection was less pronounced for the *COIII* and *ND2* genes in comparison with the rest of the PCGs. The codons encoding Leucine (CUN), Threonine, and Isolecucine were the three most frequently present. The secondary structure of rRNA of Lacertidae (herein, *E. scripta* KZL15 as an example) comprised four domains and 28 helices for 12S rRNA, with six domains and 50 helices for 16S rRNA. Five types and twenty-one subtypes of CR in Lacertidae were described by following the criteria of the presence and position of tandem repeats (TR), termination-associated sequence 1 (TAS1), termination-associated sequence 2 (TAS2), conserved sequence block 1 (CBS1), conserved sequence block 2 (CSB2), and conserved sequence block 3 (CSB3). The compositions of conserved structural elements in four genera, *Acanthodactylus*, *Darevskia*, *Eremias*, and *Takydromus*, were further explored in detail. The base composition of TAS2 – TATACATTAT in Lacertidae was updated. In addition, the motif “TAGCGGCTTTTTTG” of tandem repeats in *Eremias* and the motif ”GCGGCTT” in *Takydromus* were presented. Nucleotide lengths between CSB2 and CSB3 remained 35 bp in *Eremias* and *Darevskia*. The phylogenetic analyses of Lacertidae recovered the higher-level relationships among the three subfamilies and corroborated a hard polytomy in the Lacertinae phylogeny. The phylogenetic position of *E. nikolskii* challenged the monophyly of the subgenus *Pareremias* within *Eremias*. Some mismatches between the types of CR and their phylogeny demonstrated the complicated evolutionary signals of CR such as convergent evolution. These findings will promote research on the structure and evolution of the CR and highlight the need for more mitogenomes in Lacertidae.

## 1. Introduction

A vertebrate mitochondrial genome (mitogenome) typically consists of 37 genes, including 13 protein-coding genes (PCGs), 2 ribosomal RNAs (rRNAs), 22 transfer RNAs (tRNAs), and the major non-coding fragment of the molecule, the control region (CR) [1,2,3,4]. Specifically, the CR, the most rapidly evolving part of mtDNA, could change the structure of a mitogenome by accumulating base substitutions and indels. Meanwhile, the CR is generally the main element for extensive size variation found in animal mitogenomes [5,6,7]. Accordingly, research on the CR could provide substantial insights into the molecular evolution of mitogenomes [5,7].

Despite the advancements of sequencing technology including next-generation sequencing, related sequences of the Lacertidae family and even the order Squamata are limited. There are less than 400 complete and partial mitogenomes of the species from the order Squamata, and only about 58 of them are from the Lacertidae family in GenBank (as of March 2022). The Lacertidae family, which encompasses more than 350 species distributed in Eurasia and Africa, comprises three subfamilies, i.e., Gallotiinae, Lacertinae, and Eremiainae [8,9,10,11]; but it can also be viewed from the perspective of two subfamilies—Gallotiinae and Lacertinae, with the latter being divided into two tribes, Eremiadini and Lacertini [12,13,14,15]. Within the Eremiainae subfamily, the genus *Eremias*, which occurs in Southeast Europe, western Asia, and Central Asia as well as East Asia, has significant value in phylogenetic research, medicinal utilization, and biodiversity conservation [16,17,18]. As of March 2022, the complete mitogenomes of only 13 sequences representing 10 species in *Eremias* were available in GenBank. In addition, the utilization of these sequences has mainly focused on the announcement of mitogenome organization [19]. There are few studies regarding secondary structures of rRNAs as well as the structure and evolution of CR at the genus level within Lacertidae [4,20,21,22,23].

The CR, the longest non-coding region in animal mitochondrial DNA (mtDNA), is considered to be the most variable region of mitogenome [24]. Within the CR, the displacement loop (or D-loop), which is often synonymously used in the literature with the CR [25], is in fact a region within the CR comprising a third strand of DNA creating a semi-stable structure [26]. So far, related research on the structure and evolution of the CR in animals has mainly focused on fishes, birds, and mammals [27,28,29,30]. Research related to reptiles is relatively scarce. In addition, the research on the CR of lacertid lizards (Lacertidae) mainly focused on one species, *Lacerta dugesii* [4], which is often considered as the standard reference sequence. However, using only one sequence as a reference to the whole family, even other families, may lead to some biased results due to the relatively few mitogenomes in 2003. Additionally, several rules in different genera and even in the Lacertidae family may be dismissed [31].

In this study, five complete mitogenomes of *Eremias* were newly determined and compared with other Lacertidae mitogenomes available in GenBank. Specifically, we compared the CR of 58 taxa (with 53 complete CR among them), which represent 13 genera within Lacertidae. Overall, five general types and twenty-one subtypes of CR within Lacertidae were found. The findings, which could refine the annotations of the CR at the genus level and family level, will guide future research on the structure and evolution of the mitochondrial CR. In addition, we implemented phylogenetic analyses of 61 taxa with maximum likelihood (ML) and Bayesian inference (BI) approaches by using 13 concatenated PCGs. This study tested the phylogenetic relationships among the major lineages within Lacertidae in general, and among the subgenera of *Eremias* in particular. Above all, the results provide new insights into the structure and evolution of the mtDNA CR in lacertid lizards. Additionally, the presentation of rRNA structures could promote the application of increasingly complex sequence evolution models in maximum likelihood and Bayesian methods.

## 2. Materials and Methods

### 2.1. Sample Collection and DNA Extraction

The five specimens of *Eremias* were captured by hand in Kazakhstan and Kyrgyzstan; related information is listed in Table 1. The collection of lizards used for this study obeyed the Law “On the Animal World” No. 59 of Kyrgyzstan and followed the guidelines in the Institute of Biology and Soil, National Academy of Science of the Kyrgyz Republic, as well as those in the Institute of Zoology of Republic of Kazakhstan. 

The captured lizards were euthanized with an overdose of sodium pentobarbital delivered via intraperitoneal injection. The liver samples and voucher specimens were fixed in 95% ethanol and deposited in the Chengdu Institute of Biology (CIB), Chinese Academy of Sciences. The Animal Care and Use Committee of CIB (Permit Number: CIB-20121220A) approved all of the animal procedures.

Total genomic DNA was extracted from the liver tissue, which contained higher mitochondrial DNA content, with a rapid high-salt procedure [32]. The integrity of DNA samples was measured using 1% agarose gel electrophoresis and HiPure Universal DNA Kit (Magen Biotech, Shanghai, China). DNA concentration and purity were measured using a NanoDrop 2000 spectrophotometer (Thermo Scientific, Wilmington, DE, USA) and Qubit 2.0 Flurometer (Invitrogen, Carlsbad, CA, USA).

### 2.2. Library Construction and High-Throughput Sequencing

The sample genome DNA, which passed the test for data quality control which contained quality distribution, error rate distribution, and base distribution, was selected and then fragmented via mechanical interruption (ultrasound). Selected fragments were purified and repaired. The addition of A on 3′ end and the connection of sequencing connectors were completed. Fragments 350 bp in size were selected using the method of agarose gel electrophoresis, and PCR amplification was carried out to produce sequencing libraries which were formed using the standard procedure of Illumina DNA library construction in Genepioneer Biotechnologies Co. Ltd. (Nanjing, China). A VAHTS^®^ Universal DNA Library Prep Kit was used to construct the libraries. The qPCR method and an Agilent 2100 Bioanalyzer (Agilent, Palo Alto, CA, USA) were used for the control of the libraries’ quality. The qualified libraries were sequenced using the Illumina NovaSeq platform (Illumina, San Diego, CA, USA), with the sequencing read length PE 150 bp in Genepioneer Biotechnologies Co. Ltd. (Nanjing, China).

### 2.3. Sequence Assembly, Annotation, and Analysis

The raw data obtained via Illumina NovaSeq sequencing were filtered to obtain high-quality sequences with fastp v0.20.0 [33,34] by trimming adapters and primers and filtering reads with phred quality <Q5 and N base number >5. The obtained high-quality fragments for each sample were aligned with the *E. stummeri* mitogenome in GenBank (accession no. KT372881) to remove sequence repeats and inaccurate sequencing, and then assembled using SPAdes v3.10.1 [35,36] to obtain the complete circular mitogenome. The online BankIt software [37] was used to submit the complete mitogenomes to GenBank. NCBI BLAST [38] and MITOS [39,40] were used to identify the boundaries of PCGs and rRNAs. The potential cloverleaf structures and boundaries of tRNAs were identified using the online tRNAscan-SE software [41,42]. The secondary structures of two rRNAs were predicted using the online RNAfold software [43] and rearranged using Microsoft PowerPoint (14.0.4760.1000) without changing the structure. In addition, helix numbering of rRNAs was designated following the convention of the Comparative RNA Web (CRW) Site [44] and related studies [20,45,46]. The mitogenomic map was generated using the online OGDRAW v1.3.1 software [47,48]. Non-synonymous and synonymous substitutions of PCGs of Lacertidae and nucleotide composition were computed in MEGA v7.0 software [49]. Taxon information and the GenBank accession numbers of 61 sequences are listed in Table 2. Composition skew values were computed, utilizing the formulae: AT-skew = ((A% − T%)/(A% + T%)); GC-skew = ((G% − C%)/(G% + C%)). The boundaries and the size of CR were confirmed using the position of *tRNA^Phe^* and *tRNA^Pro^*. In addition, the sequence comparison with previously reported *Eremias* mitogenomes is another significant method. The CR composition and feature analysis were implemented in MEGA v7.0 [49]. In addition, the base distribution and relative synonymous codon usage (RSCU) values were calculated in MEGA v7.0 [49]. Tandem repeats in the CR were detected in the tandem repeats finder online server [50,51], and the results generated by the server were selected according to the copy number.

### 2.4. Phylogenetic Analysis

Mitogenomes of 53 lacertids and 3 outgroup taxa were downloaded from GenBank [75], and 5 mitogenomes of *Eremias* were determined in this study (see Table 2). The BI and ML methods were used for phylogenetic inference. Based on most recent knowledge on higher-level relationships of squamate reptiles [76,77,78], several families such as Amphisbaenidae, Bipedidae, and Rhineuridae are closely related to Lacertidae. Accordingly, *Am. schmidti* and *B. biporus* (available in GenBank) were selected as the outgroup taxon. In addition, one mitogenome representing the family Gymnophthalmidae, *Lo. percainatum,* available in GenBank (accession number MW864329), was chosen to root the tree due to its relatively distant relation to Lacertidae [76,77,78].

The concatenation of 13 PCGs and alignment of the 61 sequences were processed in MEGA v7.0 [49] with the default parameters, and then, we checked them manually. With the help of a plug-in program in PhyloSuite v1.1.16 [79], we completed gene partitioning and tree construction. Meanwhile, we selected the best partitioning schemes and evolutionary models, which were estimated using PartitionFinder v2.1.1 [80], with the greedy algorithm and corrected Akaike information criterion (AICc). In order to find the partitioning schemes models for ML and BI analyses, respectively, we utilized the “all” and “Mrbayes” modes. MrBayes v3.2.6 [81,82] was utilized for partitioned Bayesian analyses, with four independent runs for two million generations and sampling every hundred generations. The convergence of the independent runs was assessed by checking the effective sample size (ESS) >200 calculated in Tracer v1.7.1 [83], and the average standard deviation of split frequencies <0.01. A 50% majority-rule consensus tree and the posterior probability (PP) of clades were assessed by combining the sampled trees from the two independent runs after a 25% burn-in phase. We interpreted PP ≥ 0.95 to be strongly supported [84,85]. The information concerning best-fit substitution models and partitioning schemes for PCGs is listed in Table S1. IQ-TREE v1.6.7 [86] was used to construct the ML tree. We used an ultrafast bootstrap approximation approach with 5000 bootstraps. Nodes with UFBoot ≥ 95 were considered to be well-supported [87]. We also computed the uncorrected pairwise distance (p-distance) among species in *Eremias* with MEGA v7.0 [49]. In the end, FigTree v1.4.3 [88] was used for the tree visualization, and Microsoft PowerPoint (2010) was used for the tree edits.

## 3. Results 

### 3.1. Genome Organization and Base Composition

**Figure 1:**
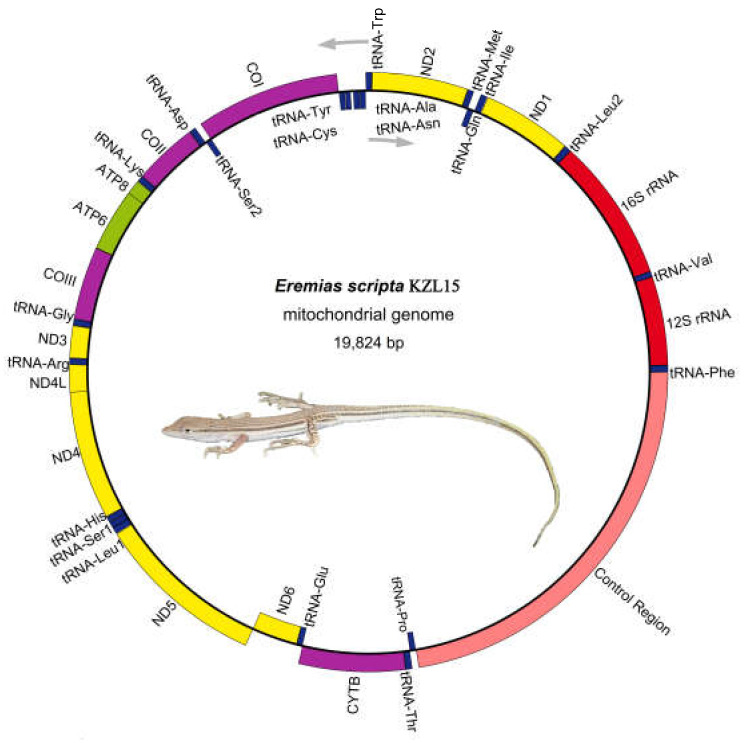
Mitochondrial genome map of *E. scripta* KZL15. Genes encoded by the light or heavy strand are indicated inside or outside, respectively, showing the direction of transcription. The tRNAs are denoted by color blue and labeled according to the three-letter amino acid codes. The mitogenome maps of *E. scripta* KZL44, *E. nikolskii*, *E. szczerbaki,* and *E. yarkandensis* are similar to that of *E. scripta* KZL15. Lizard photo by Xianguang Guo.

The mitochondrial genome of *E. scripta* KZL15 (19,824 bp), *E. scripta* KZL44 (19,831 bp), *E. nikolskii* (20,840 bp), *E. szczerbaki* (19,650 bp), and *E. yarkandensis* (18,743 bp) were sequenced, annotated, and compared with the other 53 taxa of Lacertidae in several aspects. The composition and the arrangement of mitochondrial genes in these species were the same as those in most other typical vertebrates (Figure 1, Table S2). They all consisted of 13 PCGs, 22 tRNA genes, 2 rRNA genes (12S rRNA and 16S rRNA), and 2 non-coding regions (the CR and origin of replication on the light-strand (O_L_)). The length of O_L_ ranged from 27 bp to 30 bp of the five racerunners. We found that the O_L_ motifs of *E. scripta* KZL15, *E. scripta* KZL44, and *E. nikolskii* were relatively similar; they all contained the same composition, 5′-”TTCCCCCGTTANNNNNAAAACGGGGG”-3′. Additionally, *E. szczerbaki* and *E. yarkandensis* both contained the O_L_ motif 5′-”TTCCCCCGTTANNNNAAAACGGGGG”-3′, and there was just one base difference between the two O_L_ motifs. Most genes (12 PCGs, 2 rRNAs, and 14 tRNAs) were distributed on the H-strand, while 9 genes (*ND6* and 8 tRNAs) were encoded on the L-strand.

The nucleotide composition, AT skew, and GC skew of total mitogenomes, PCGs, rRNAs, tRNAs, and CR of 58 taxa in the Lacertidae were calculated. The mean AT nucleotide content of the five complete mitogenomes was nearly similar: 61.33% in *E. scripta* KZL15, 61.16% in *E. scripta* KZL44, 58.54% in *E. nikolskii*, 59.47% in *E. szczerbaki,* and 59.46% in *E. yarkandensis*. Additionally, the nucleotide and composition skew values were conserved in the family Lacertidae (Table S3); they all showed a positive AT-skew (0.043 to 0.053) and a negative GC-skew (−0.351 to −0.324), suggesting a strong AT bias, and the AT content was higher in the CR (from 57.6% to 76.7%).

### 3.2. PCGs and Codon Usage

**Figure 2:**
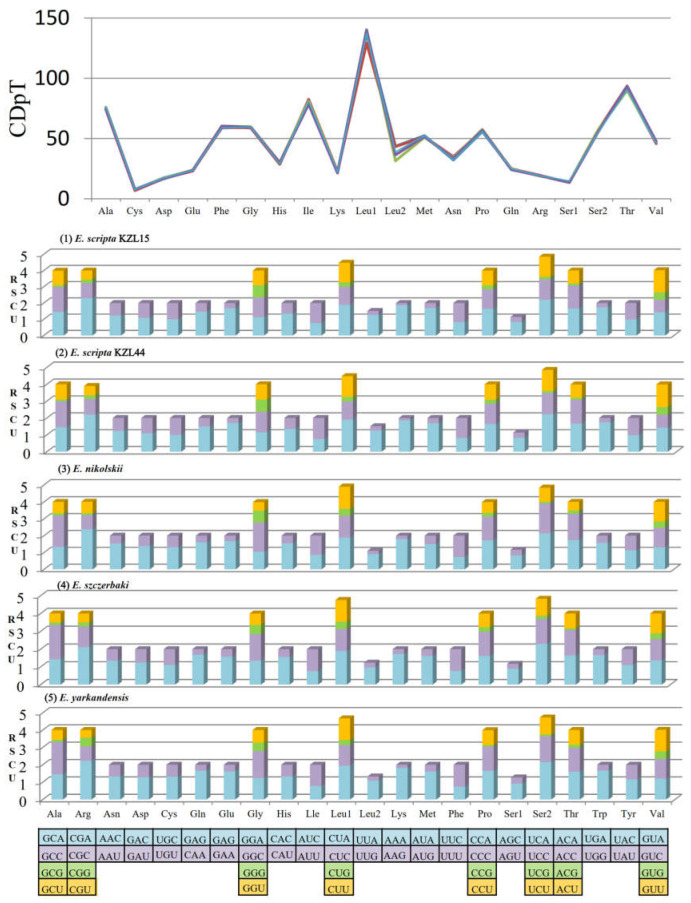
The base composition and the relative synonymous codon usage (RSCU) values of *E. scripta* KZL15, *E. scripta* KZL44, *E. nikolskii*, *E. szczerbaki*, and *E. yarkandensis*, respectively. CDpT stands for codons per thousand codons. The color of blue, purple, green and yellow means the first, second, third and fourth type of each amino acid.

All newly sequenced *Eremias* mitogenomes contained 13 PCGs (*ND1-ND6*, *ND4L*, *ATP8*, *ATP6*, *CYTB*, and *COI−COIII*) ranging from 62 bp (*ATP8*) to 1824 bp (*ND5*). The total length of PCGs of the five racerunners ranged from 11,373 bp (*E. yarkandensis*) to 11,375 bp (*E. nikolskii* and *E. szczerbaki*). The start codons of 12 PCGs of *E. scripta* KZL15, *E. scripta* KZL44, *E. nikolskii,* and *E. szczerbaki* were ATG, whereas COI in the five racerunners and *ND1* in *E. yarkandensis* showed the start codon GTG. In addition, there were five typical types of stop codons containing three canonical (TAA, TAG, and AGG) and two truncated stop codons (TA– – and T– –). By comparing the PCGs of 58 taxa in Lacertidae, we found two conserved motifs of the overlap, i.e., “ATGGNNNTAA” and “ATGANTAA” between *ATP8* and *ATP6*. The overlap between *ATP8* and *ATP6* kept 10 bp in Lacertidae. More interestingly, the completely identical overlap motif “ATGGNNNTAA” was only presented across all species of *Eremias*. In addition, we compared the same structure of several other families of different orders in vertebrate, such as fishes in the subfamily Cobitinae [89], and found that the overlap composition of the majority of vertebrates contained the motif “ATGGNNNTAA”; the base of “NNN” may be their genus-specific feature and even family-specific feature. We also found the length of overlaps between *ND4* and *ND4L* (7 bp), *ND5* and *ND6* (5 bp), *ATP6* and *COIII* (1 bp) were consistent in the five racerunners. The relative synonymous codon usage (RSCU) and codon distribution of the five *Eremias* mitogenomes were analyzed (Figure 2). The total number of codons of the five racerunners was similar: 3790 in *E. scripta* KZL15, *E. nikolskii,* and *E. szczerbaki*; 3789 in *E. scripta* KZL44 and *E. yarkandensis*. The codon distribution among the five racerunners was coincident; the codons encoding Leucine (CUN), Threonine, and Isolecucine were the three most frequently present, while Cysteine was the rarest of them. In addition, the patterns of five racerunners were also consistent with one another. The codons were biased to utilize more A/U than G/C at the end, which resulted in the content of AT being higher than GC in the third position of *Eremias* PCGs.

### 3.3. Transfer RNAs and Ribosomal RNAs

The tRNA secondary structure and strand bias were coincident among the five racerunners and even in other lizards [31]. Among the 22 tRNA genes, only *tRNA^ser(AGN)^* (Ser1) and *tRNA^cys^* could not be folded into a typical cloverleaf secondary structure and had no recognizable DHU arm (Figure S1). The length of single tRNA gene varied from 62 bp to 73 bp, and the total length of 22 tRNA genes ranged from 1513 bp to 1517 bp in the five *Eremias* mitogenomes.

**Figure 3:**
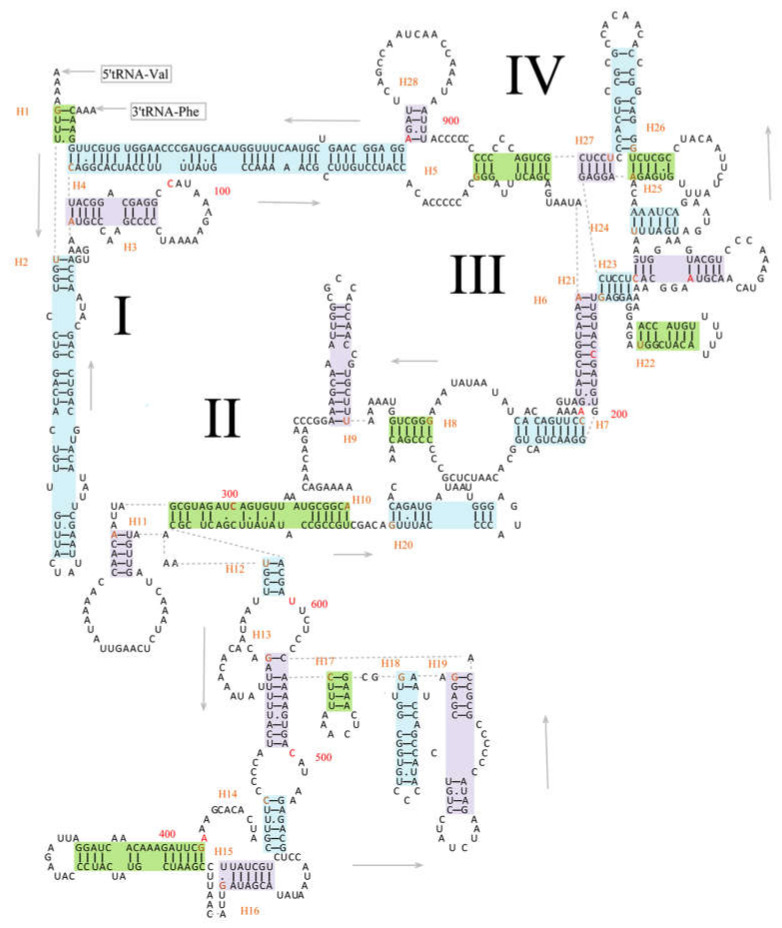
Predicted secondary structure of the 12S rRNA in *E. scripta* KZL15. Watson-Crick base pairings are indicated by the dashes (–); G–U base pairings are indicated by dots (•). Roman numerals indicate the conserved domain structures; I–IV indicate four domains in the secondary structure of 12S rRNA. Every 100th base is marked in red. Helices were marked with green, purple, and blue. The number of helices and the first base of helix were marked in orange; H means helix. Generated using RNAfold [43] and edited using Microsoft PowerPoint (14.0.4760.1000).

**Figure 4:**
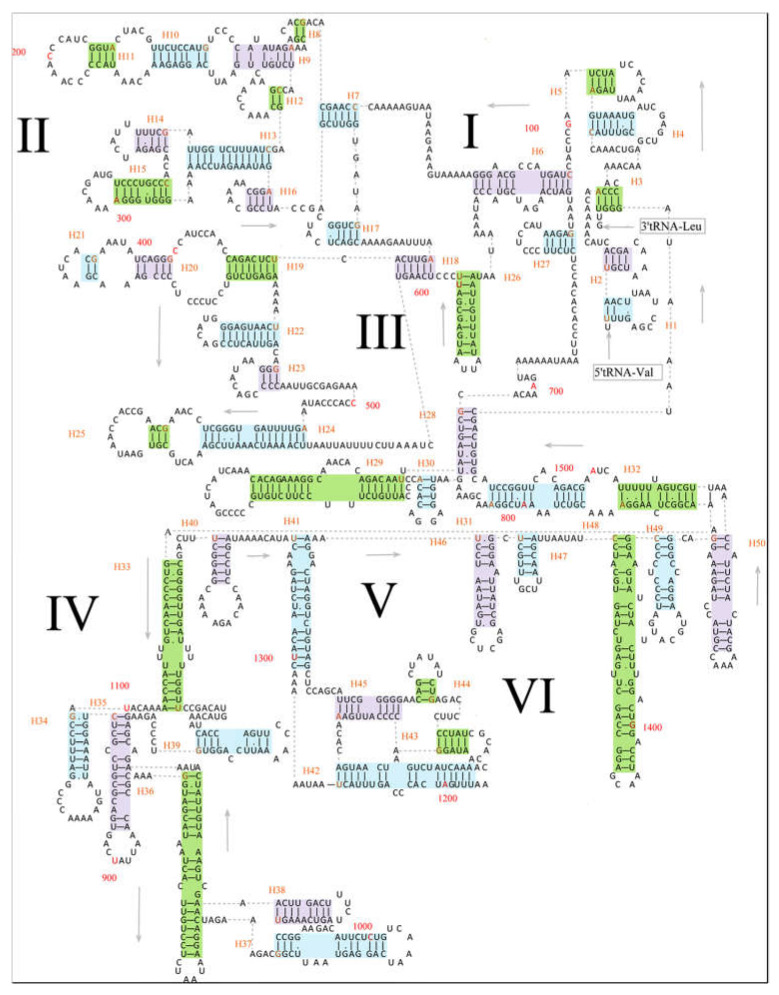
Predicted secondary structure of the 16S rRNA in *E. scripta* KZL15. Watson-Crick base pairings are indicated by the dashes (–); G–U bases pairings are indicated by dots (•). The numbering of helix follows Hickson et al. [45]. Roman numerals indicate the conserved domain structures; I–VI indicate six domains in the secondary structure of 16S rRNA. Every 100th base is marked in red. Helices were marked with green, purple, and blue. The number of helices and the first base of helix were marked in orange; H means helix. Generated using RNAfold [43] and edited using Microsoft PowerPoint (14.0.4760.1000).

The 12S rRNA and 16S rRNA were located between *tRNA^Phe^* and *tRNA^Leu2^* genes and interposed by *tRNA^Val^*. The length of 12S rRNA of the five specimens was 951 bp; however, the length of their 16S rRNA was variable, 1542 bp in *E. scripta* KZL15 and *E. scripta* KZL44, 1545 bp in *E. nikolskii*, 1537 bp in *E. szczerbaki*, and 1543 bp in *E. yarkandensis*. With reference to previous studies on secondary structures of 12S rRNA [31,45], we defined four domains of 12S rRNA of *E. scripta* KZL15 (as an example of Lacertidae) and defined 28 helices which contained 18 GU pairs. The pairing of guanine and uracil was permitted, in consideration of the structurally stable structure in RNAs [90]. As illustrated in Figure 3, Domain I contained helices 1–5; Domain II contained helices 11–19; Domain III contained helices 6−10 and helix 20; Domain IV contained helices 21–28. Of these 28 helices, helix 6 was the most stable one with fewer bulges and internal loops, while helix 4 was the most variable one with more unpaired bases. Meanwhile, according to the 16S rRNA of *Darevskia* genus within the Lacertidae from Brown in 2005 [20], we defined six domains of 16S rRNA of *E. scripta* KZL15, which contained 50 helices (Figure 4). Domain I consisted of helices 1−6 and helix 27; Domain II consisted of helices 8−16 and helices 19−24; Domain III contained helices 17–18 and helices 26–32; Domain IV included helices 33−44; Domain V contained helices 41–45, and Domain VI consisted of helices 46−50. Thirty GU pairs were found among the six domains. Meanwhile, we detected the most stable stem (helix 26) and the most variable structure (helix 48) for 16S rRNA. 

### 3.4. Non-Synonymous and Synonymous Substitutions

**Figure 5:**
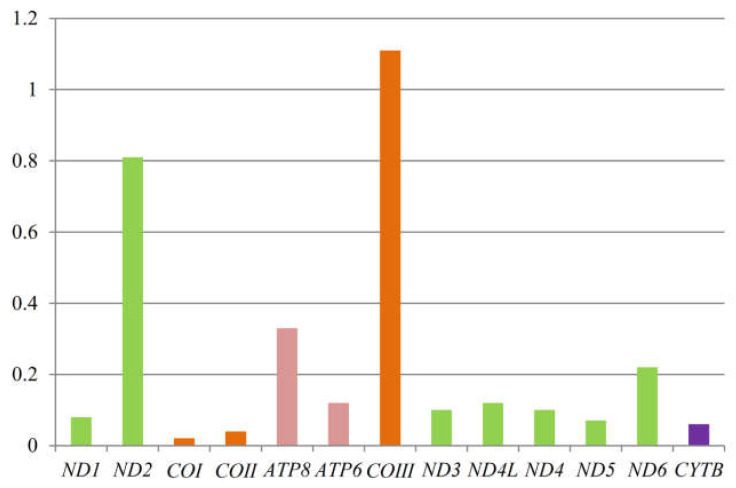
The nonsynonymous/synonymous ratios (dN/dS) in 13 mitochondrial PCGs of 58 taxa in Lacertidae. The histogram represents the average dN/dS for each PCG.

To further understand the role of selective pressure and the evolution of Lacertidae, we computed the average dN/dS value of each PCG of 58 taxa. We found that the Ka/Ks values for all PCGs except *COIII* were lower than one (between 0.02 and 0.81) (Figure 5), indicating that they are evolving under purifying selection. Among the 13 protein-coding genes, the average dN/dS of *COIII* was the highest (1.11), and *ND2* (0.81) also had very high average dN/dS values. 

### 3.5. Structure of Control Region

Many studies have demonstrated that the CR in vertebrates shows a similar structure and conserved sequences [7,27,28,91,92], indicating evolutionary constraints and conservatism at various levels. We presented the composition of conserved structure elements of the CR in 58 taxa representing 13 genera in Lacertidae. Overall, there were more mitogenomes from four genera, *Acanthodactylus, Eremias*, *Takydromus,* and *Darevskia,* than those from other genera available in GenBank. We also analyzed the TR of CR in Lacertidae and found several motifs of *Eremias* and *Takydromus*. The CR was longest in *E. nikolskii* (5436 bp) and shortest in *Al. nigropunctatus* (146 bp).

Conserved structural elements of 58 lacertid taxa were analyzed and compared with the reference species of *L. dugesii*, which contained one or two termination-associated sequences (TASs) and three conserved sequence blocks (CSBs). The position of conserved structural elements is listed in Table S4. Overall, the compositions of TAS1, CSB1, and CSB2 were consistent with those of the reference sequence (Table 3, Table 4 and Table 5). Compared to the TAS1 (ACTATTATGTATATAGTGCATTAA) of *L. dugesii*, TAS1 of six species of *Eremias* and five species of *Acanthodactylus* were similar to one another, showing “·················A······” (see Type 2 in Table 3) (dots indicate the same base as the reference motif). The CSB1 of 12 species exhibited the standard motif “CTATATGGTATTATTGTCTTAATGCTTGGTAGACATAT” (see Type 1 in Table 4), which was used to compare and determine the presence of CSB1 of CR in the family Lacertidae and even reptiles, whereas this structure in the other 13 species was “···············C·T····················” (see Type 2 in Table 4). As such, the composition of CSB1 in Lacertidae warrants further study. As shown in Table 6, the TAS2 of only one species (*L. viridis viridis*) was similar to that (CATACATTAA) of *L. dugesii*, and the structures of 13 species were “T··G······“ (see Type 2 in Table 6). Additionally, the first base of 29 species was T; thus, the TAS2 of the standard reference sequence, which was traditionally used to compare and determine the presence of TAS2 of the CR in Lacertidae and even reptiles, should be updated to “TATGCATTAA”.

On the basis of the comparisons, we defined the standard compositions of TAS1, TAS2, and CSB1 in *Eremias*. In addition, there were also several genus-specific features of these three conserved structures in *Takydromus* and *Darevskia*. The TAS1 in *Darevskia* and CSB1 in *Takydromus* were exactly similar to that in the reference species *L. dugesii*. However, the TAS2 “T··G······” and CSB1 ”···············C·T····················” in *Darevskia* were specific at the genus level. Generally, CSB2 and CSB3 are conserved in the family Lacertidae [4,21,93], and they are very conserved in the four compared genera (Table 5, Table 6 and Table 7). For *Eremias* and *Darevskia*, the length between CSB2 and CSB3 kept 35 bp. The compositions of conserved structures of the CR in Lacertidae are listed in Table S5.

Tandem repeats are one of the factors accountable for extensive size variations in mitogenomes [7,92,93]. Tandem repeats were reported in the CR of several lacertid lizards [4,21,25]. In this study, we also compared the TR of 46 lacertids (see Table S6 for details), which contained the copy number, length, and motif information of TR. There are also several genus-specific features. The motif segment “TAGCGGCTTTTTTG” was present in the 11 examined species of *Eremias*. The motif segment “GCGGCTT” was present in the seven examined species of *Takydromus* excluding *T. amurensis*, while six species of *Takydromus* excluding *T. wolteri* showed the motif segment “TTTTCC”. Compared to these genera, the feature of TR of *Darevskia* was relatively weaker, and the motif segment “CAAAACTTTTAA” was present in just 9 of the 17 examined species. We found the position between TR and TAS1 of most species of *Eremias*, *Takydromus*, and *Lacerta* were conserved; there was one TR before TAS1 of these genera. The position between the TR and CSB1 was conserved in *Darevskia*; there was a TR located between CSB1 and CSB2. We also found the position between the TR and CSB3 were conserved in *Eremias*; there was one TR after CSB3. Overall, the composition and position of TR/TAS/CSB in *Eremias* was markedly conserved.

**Figure 6:**
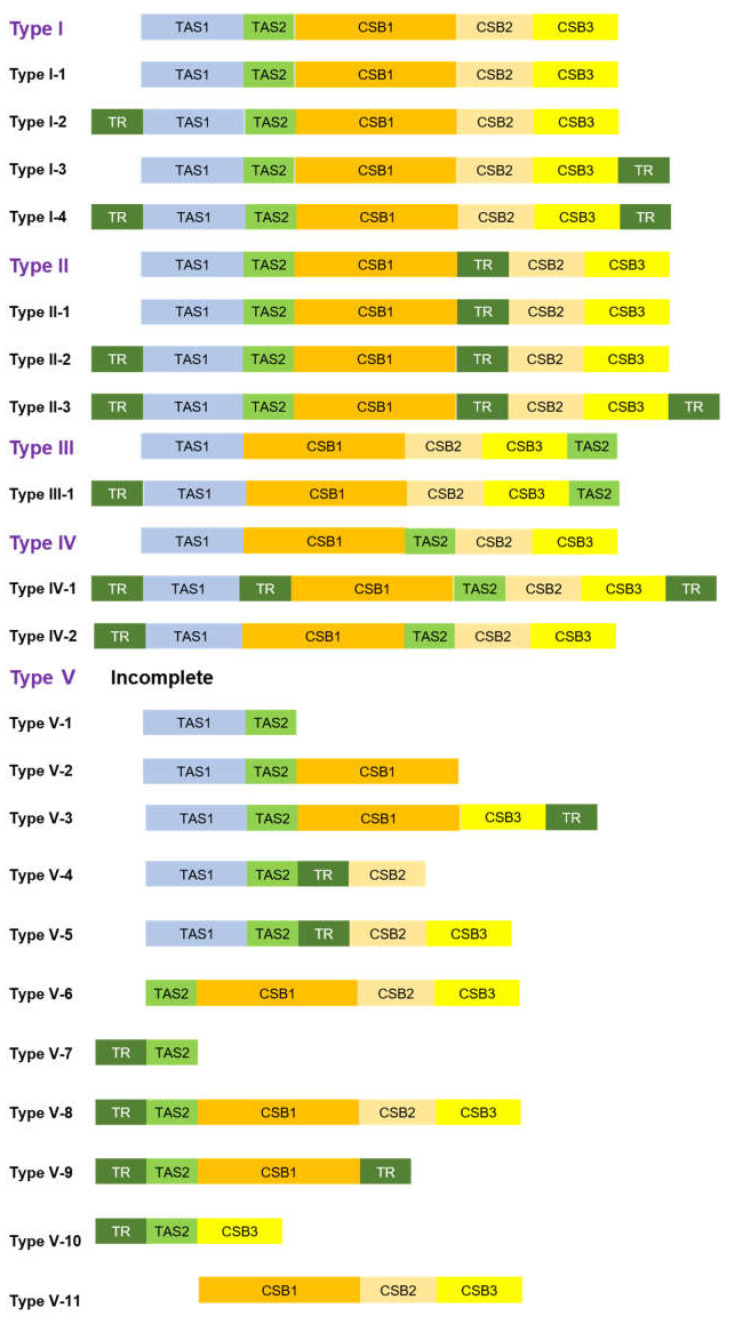
Types of the control region in Lacertidae, with consideration of positions of TAS1, TAS2, CSB1, CSB2, CSB3 and TR. Type I-1: *Ac. aureus*, *Ac. schmidti*, *D. valentini*, *Mes. olivieri*; Type I-2: *Ph. kulzeri*, *Po. siculus*, *Z. vivipara*, *D. chlorogaster*; Type I-3: *Pe. laticeps*; Type I-4: *E. multiocellata*, *E. stummeri*, *E. przewalskii*, *E. yarkandensis*, *E. szczerbaki*, *E. dzungarica*, *E. nikolskii*, *E. argus*; Type II-1: *D. dahli*, *D. parvula*, *D. portschinskii*, *D. rudis*, *D. saxicola*, *L. bilineata*, *Ac. boskianus*, *Ps. algirus*; Type II-2: *D. armeniaca*, *D. brauneri*, *D. daghestanica*, *D. mixta*, *D. unisexualis*, *L. agilis*, *L. viridis*, *Po. muralis*; Type II-3: *E. scripta*; Type III-1: *T. septentrionalis*, *T. wolteri*, *D. derjugini*, *T. kuehnei*, *T. sylvaticus*, *D. raddei*; Type IV-1: *E. brenchleyi*; Type IV-2: *D. chlorogaster*; Type V-1: *Al. nigropunctatus*; Type V-2: *Ac. guineensis*; Type V-3: *E. vermiculata*; Type V-4: *Ac. erythrurus*; Type V-5: *Au. australis*; Type V-6: *D. praticola*; Type V-7: *T. sexlineatus*; Type V-8: *T. amurensis*; Type V-9: *D. caucasica*; Type V-10: *D. clarkorum*; Type V-11: *Mer. squamulosus*.

**Figure 7:**
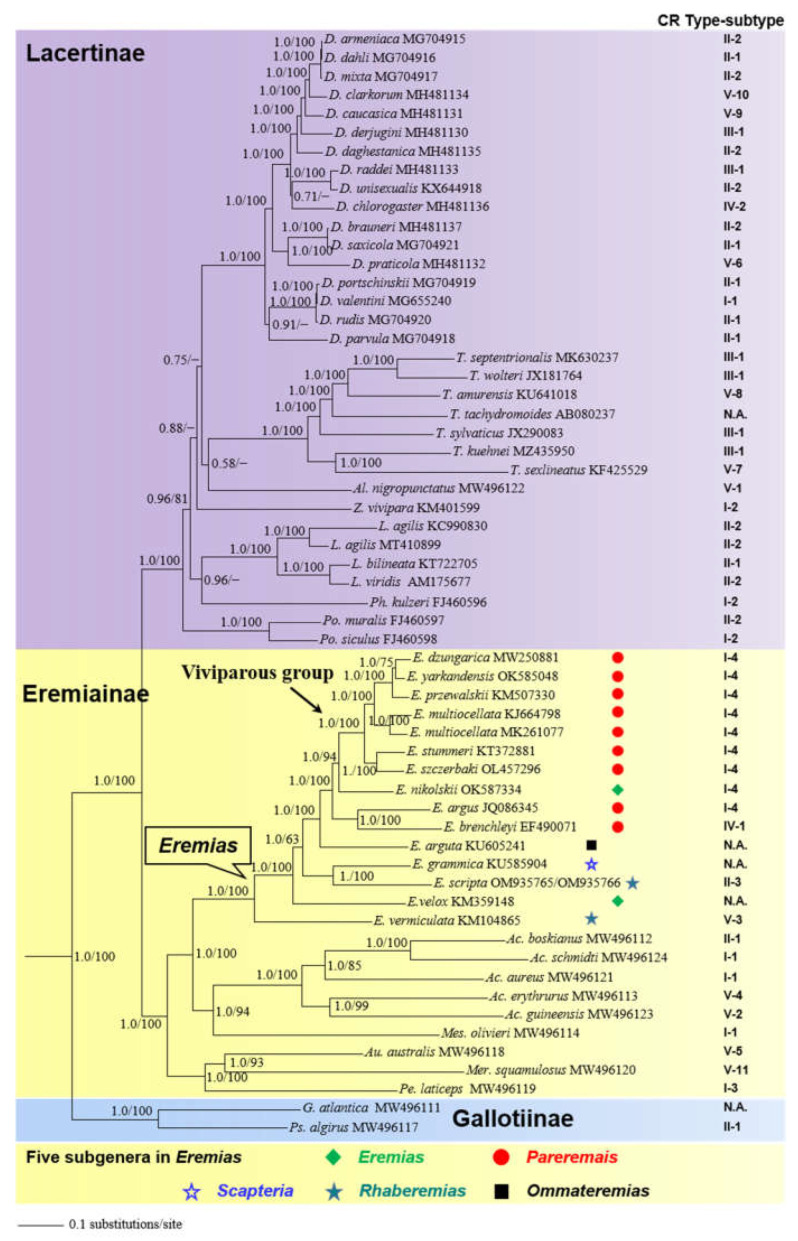
A 50% majority-rule consensus tree of the family Lacertidae inferred from partitioned Bayesian analyses based on the concatenated PCGs of 58 lacertids and 3 outgroup taxa (not shown for clarity). Node numbers indicate Bayesian posterior probabilities (PP) and ML ultrafast bootstrap values (UFBoot), respectively. Dashes represent nodes with bootstrap support lower than 50% or represent nodes that do not exist in ML tree. Branch lengths represent means of the posterior distribution. GenBank accession numbers are given with species names, and subfamily assignments are listed, along with the viviparous group/subgenera in *Eremias*. Types/subtypes of the CR are mapped on the tree and correspond to those depicted in Figure 6. N.A., not applicable, for species with incomplete CR available in GenBank.

According to the criteria of the presence and position of TAS1, TAS2, CBS1, CSB2, and CSB3, as well as the anormal TR which is located between the conserved structure elements, we found five general types of the CR (Figure 6). Meanwhile, based on the criteria of general types, we further considered the presence and position of a normal TR, which is located outside the conserved structure elements, as the additional condition, and found a total of 21 subtypes of CR from Lacertidae (Figure 6). Notably, a close link between the types/subtypes of CR from some species of Lacertidae and their phylogeny can be observed (see Figure 7 for details). 

### 3.6. Phylogenetic Analysis

Bayesian inference and ML analyses produced highly congruent topology, with only minor differences on some nodes in the subfamily Lacertinae. Thus, only the BI tree with both PP and UFBoot from ML is presented (Figure 7), and see Figure S2 for ML tree. With limited taxon sampling, the monophyly of Lacertidae was recovered with strong support (PP = 1.0; UFBoot = 100), in accord with previous studies [9,10,76]. Moreover, the resulting trees confirmed a sister relationship between Eremiainae and Lacertinae with strong support (PP =1.0; UFBoot =100) [8,12]. In the subfamily Lacertinae, the phylogenetic position of most genera was unresolved due to lower support (PP < 0.95; UFBoot < 50). In the subfamily Eremiainae, the phylogenetic position of six sampling genera was resolved, with *Eremias* being more closely related to *Acanthodactylus* + *Mesalina* (PP =1.0; UFBoot =100). Within *Eremias*, the monophyly of the viviparous group was recovered with strong support (PP = 1.0; UFBoot = 100), which is consistent with previous studies [16,18,94]. However, *E. nikolskii* was inferred as the sister taxon to the viviparous group with strong support (PP = 1.0; UFBoot = 94). In addition, as demonstrated by p-distances (Table S7), the genetic divergence between *E. nikolskii* and the viviparous group (0.105–0.109) was smaller than that of *E. arugs-E. brenchleyi* versus the viviparous group (0.122–0.124). 

## 4. Discussion

### 4.1. Secondary Structures of rRNA Are Useful for Phylogenetic Inference

In the present study, the secondary structures of 12S rRNA and 16S rRNA in *E. scripta* KZL15, as a representative lacertid, were presented in detail for the first time (see Figure 3 and Figure 4). Considering these secondary structural features, rRNA can be divided into paired (stem) and unpaired (loop) regions. Compensatory substitutions occur frequently in the paired regions; the property contradicts the assumption of independent mutations [95,96]. The analysis of RNA secondary structures is helpful to aid the alignment of rRNA sequences [97] and contributes to the increasingly sophisticated models of sequence evolution being applied in maximum likelihood and Bayesian approaches [20,98]. For instance, the doublet model of MrBayes [81,82] is intended for stem regions of ribosomal sequences, where nucleotides pair with each other to form doublets. There are various ways to model the evolution of nucleotide doublets. One method is to focus on the common doublets, A-T and C-G in particular. MrBayes uses a more complex model, originally formulated by Schöniger and von Haeseler [99], where all doublets are taken into account. Accordingly, the phylogenetic performance of the rRNA can be improved by incorporating information regarding its secondary structure in analyses for more accurate phylogenetic inference [84,100,101,102]. We believe that the secondary structure information of rRNAs described herein is useful for phylogenetic inference among species in Lacertidae.

### 4.2. Selection Pressure on PCGs

The phenomenon of purifying selection of PCGs is usually detected in most Metazoa [91]. As shown in Figure 5, the values of all PCGs except *COIII* were smaller than 1, which can be interpreted as meaning that the proteins evolve slowly under purifying selection, i.e., are more conserved [103,104]. This may be explained by the rationale that most of the nonsynonymous substitutions are detrimental to fitness and consequently have low fixation probabilities. A possible reason for the less extent of purifying selection of *ND2* is that relaxed purifying selection drives the evolution of *ND2* by mostly affecting regions that have lower functional relevance [105]. The *COI* gene showed the lowest value (0.02), and *COIII* showed the highest value (1.11). With the ratio dN/dS > 1, *COIII* may be considered under positive selection [103,104]. Further research is necessary to detect the variation of selective pressures among different lacertid lineages and to quantify the probability of positive selection on each site in each gene across all lacertids.

### 4.3. Phylogenetic Implications

Phylogenetic analyses based on 13 concatenated PCGs statistically recovered the higher-level relationships among the three subfamilies in Lacertidae (Figure 7). As for the subgenus assignment in *Eremias*, we acknowledge the plenary powers of the International Code on Zoological Nomenclature (ICZN) to designate *Lacerta velox* Pallas, 1771, as the type species of *Eremias* [106]. As such, the subgenera *Eremias* and *Rhaberemias* are not monophyletic, which is also congruent with previous results [16,17]. The phylogenetic position of *E. nikolskii* and its genetic affinity demonstrated by p-distances together challenged the monophyly of the subgenus *Pareremias* [16,18]. Nevertheless, the phylogenetic position of most genera in the subfamily Lacertinae was unresolved (PP < 0.95; UFBoot < 50), corroborating the hypothesis of a hard polytomy in the Lacertinae phylogeny due to fast radiation [10,14].

### 4.4. Structure and Evolution of Control Region

On the one hand, due to a lack of typical coding constraints, the CR is usually thought to be the fastest evolving region of the mitogenome [27], so it is broadly utilized to infer intraspecific and interspecific phylogenetic relationships. On the other hand, the CR constrains sequences related to the termination of H-strand replication, the origin of H-strand, and promoters of transcription to both L- and H-strand [7,28,107,108,109]. This indicates that the CR has evolutionary constraints. Indeed, many studies have demonstrated that the CR in vertebrates shows a similar structure and conserved sequences [7,27,28,91,92], indicating evolutionary constraints and conservatism at various levels. In addition, many CSBs identified suggest that many unknown functions exist. It is these known and unknown functions that put the CR under high evolutionary pressure and may lead to this conservation.

The mitochondrial control region had not been considered as a transcriptional region until 2018, when Gao et al. [110] documented that this region encodes two long non-coding RNAs (lncRNAs). However, current methods of the annotation of animal mitogenomes are still limited to blastx or structure-based covariance models [39]. Thus, it is necessary to further use a small RNA sequencing (sRNA-seq)-based method [111,112] to obtain improved annotations of the lacertids mitogenome at 1 bp resolution and to decipher TR in the CR.

By mapping the trait on the tree, we found that the CR from *Mes. olivieri* and the majority of species in *Acanthodactylus* and *Eremias* belonged to Type I, showing a close link with their phylogeny. Additionally, the CR from most species in *Darevskia* and species in *Lacerta* belonged to Type II; these species also presented closer affinities on the tree. The CR from minority species in *Darevskia* and four species in *Takydromus* belonged to Type III, demonstrating, to some extent, a close link with their phylogeny. Several similar, almost completely clade-specific insert and tandem repeat signatures were detected in the *Lacerta viridis* complex [23]. On the other hand, convergent evolution may be attributed to the mismatch for there not to be subfamily- or genus-specific types of the CR. In a framework of subtypes, we found several mismatches with phylogeny; this may imply the complexity of evolution in the CR. For example, in Type I-1, two species of *Acanthodactylus* and *Mes. olivieri* presented a closer relationship; however, *D. valentini* was somewhat distantly related to them. This pattern was also mirrored in Type I-2 and Type II-1. In addition, with regard to types of CR, several species also presented some independent evolutionary scenarios, such as *E. argus*, *D. valentini*, and *D. chlorogaster*, where no close link was observed between the types of CR and their phylogenetic positions. In other words, their CR types were different from those in most congeners; this phenomenon also reflected the complicated evolution of the CR. To decipher the evolutionary processes that drive the diversification of the CR in Lacertidae, further study is necessary to investigate the dynamics of the CR based on phylogenetic comparative methods in an explicit phylogenetic framework.

## 5. Conclusions

We comprehensively compared the complete mitochondrial genomes of five racerunners of *Eremias* (*E. scripta* KZL15, *E. scripta* KZL44, *E. nikolskii*, *E. szczerbaki*, and *E. yarkandensis*) for the first time. Additionally, the nucleotide composition and skew values as well as other characterizations of the mitogenomes in Lacertidae available in GenBank were comparatively analyzed. In addition, the secondary structures of 12S rRNA and 16S rRNA in *E. scripta* KZL15, as a representative lacertid, were presented in detail for the first time. Specifically, the tandem repeats, structure, and evolution of the control region from 58 taxa of Lacertidae were systematically analyzed for the first time. We found the reliable composition of TAS2 and the controversial composition of CSB1 in Lacertidae. Five general types and twenty-one subtypes of the CR in Lacertidae were unraveled. Meanwhile, we refined the composition of conserved structural elements at the genus level and found the motifs of tandem repeats in four genera, *Acanthodactylus*, *Eremias*, *Takydromus*, and *Darevskia*. Phylogenetic analyses recovered the higher-level relationships among the three subfamilies in Lacertidae and corroborated the hypothesis of a hard polytomy in the Lacertinae phylogeny due to fast radiation. *E. nikolskii* was inferred as the sister taxon to the viviparous group within *Eremias*; this challenged the monophyly of the subgenus *Pareremias*. Specifically, we found some close links of types of the CR and phylogeny, as well as some mismatches between them, which further verified the complexity of the evolutionary pattern of CR. In addition, our refinement of the secondary structures of rRNAs could promote the application of increasingly complex sequence evolution models in maximum likelihood and Bayesian approaches.

## Figures and Tables

**Table 1:** List of collection information for five racerunners in *Eremias*.

Species	Voucher Number	Collection Date	Collection Site
*E. scripta*	KZL15	July 2014	Kazakhstan; 45.91764° N, 79.96855° E
*E. scripta*	KZL44	July 2014	Kazakhstan; 45.91764° N, 79.96855° E
*E. nikolskii*	Guo4717	August 2014	Kyrgyzstan; 41.38750° N, 73.93999° E
*E. szczerbaki*	Guo4719	August 2014	Kyrgyzstan; 41.48393° N, 75.97943° E
*E. yarkandensis*	Guo4722	August 2014	Kyrgyzstan; 39.64809° N, 73.86512° E

**Table 2:** Taxon information of Lacertidae and three outgroup species analyzed in this paper with GenBank accession numbers.

Taxon	Family	Subfamily	Accession Number	Length (bp)	Reference
*Eremias*					
*E. scripta* KZL15	Lacertidae	Eremiainae	OM935765	19,824	This study
*E. scripta* KZL44	Lacertidae	Eremiainae	OM935766	19,831	This study
*E. nikolskii*	Lacertidae	Eremiainae	OK587334	20,840	This study
*E. szczerbaki*	Lacertidae	Eremiainae	OL457296	19,650	This study
*E. yarkandensis*	Lacertidae	Eremiainae	OK585048	18,743	This study
*E. dzungarica*	Lacertidae	Eremiainae	MW250881	19,899	[19]
*E. przewalskii*	Lacertidae	Eremiainae	KM507330	18,225	[52]
*E. stummeri*	Lacertidae	Eremiainae	KT372881	19,602	[53]
*E. vermiculata*	Lacertidae	Eremiainae	KM104865	19,914	[54]
*E. brenchleyi*	Lacertidae	Eremiainae	EF490071	19,542	[55]
*E. multiocellata*	Lacertidae	Eremiainae	KJ664798	18,996	[56]
*E. multiocellata*	Lacertidae	Eremiainae	MK261077	17,333	[57]
*E. argus*	Lacertidae	Eremiainae	JQ086345	18,521	[58]
*E. arguta*	Lacertidae	Eremiainae	KU605241	15,192	[59]
*E. grammica*	Lacertidae	Eremiainae	KU585904	15,338	[59]
*E. velox*	Lacertidae	Eremiainae	KM359148	18,033	[60]
*Acanthodactylus*					
*Ac. aureus*	Lacertidae	Eremiainae	MW496121	15,756	[15]
*Ac. boskianus*	Lacertidae	Eremiainae	MW496112	17,143	[15]
*Ac. erythrurus*	Lacertidae	Eremiainae	MW496113	16,827	[15]
*Ac. guineensis*	Lacertidae	Eremiainae	MW496123	16,963	[15]
*Ac. schmidti*	Lacertidae	Eremiainae	MW496124	16,943	[15]
*Australolacerta*					
*Au. australis*	Lacertidae	Eremiainae	MW496118	17,019	[15]
*Meroles*					
*Mer. squamulosus*	Lacertidae	Eremiainae	MW496120	16,860	[15]
*Mesalina*					
*Mes. olivieri*	Lacertidae	Eremiainae	MW496114	16,899	[15]
*Pedioplanis*					
*Pe. laticeps*	Lacertidae	Eremiainae	MW496119	17,046	[15]
*Algyroides*					
*Al. nigropunctatus*	Lacertidae	Lacertinae	MW496122	15,844	[15]
*Darevskia*					
*D. armeniaca*	Lacertidae	Lacertinae	MG704915	17,521	[61]
*D. brauneri*	Lacertidae	Lacertinae	MH481137	16,976	[61]
*D. caucasica*	Lacertidae	Lacertinae	MH481131	16,343	[61]
*D. chlorogaster*	Lacertidae	Lacertinae	MH481136	17,479	[61]
*D. clarkorum*	Lacertidae	Lacertinae	MH481134	16,301	[61]
*D. dahli*	Lacertidae	Lacertinae	MG704916	17,528	[61]
*D. daghestanica*	Lacertidae	Lacertinae	MH481135	17,189	[61]
*D. derjugini*	Lacertidae	Lacertinae	MH481130	16,960	[61]
*D. mixta*	Lacertidae	Lacertinae	MG704917	17,532	[61]
*D. parvula*	Lacertidae	Lacertinae	MG704918	17,510	[61]
*D. portschinskii*	Lacertidae	Lacertinae	MG704919	17,529	[61]
*D. praticola*	Lacertidae	Lacertinae	MH481132	16,418	[61]
*D. raddei*	Lacertidae	Lacertinae	MH481133	20,478	[61]
*D. rudis*	Lacertidae	Lacertinae	MG704920	17,534	[61]
*D. saxicola*	Lacertidae	Lacertinae	MG704921	17,524	[61]
*D. unisexualis*	Lacertidae	Lacertinae	KX644918	21,433	[61]
*D. valentini*	Lacertidae	Lacertinae	MG655240	17,393	[61]
*Lacerta*					
*L. agilis*	Lacertidae	Lacertinae	KC990830	17,090	[62]
*L. agilis*	Lacertidae	Lacertinae	MT410899	17,069	[63]
*L. bilineata*	Lacertidae	Lacertinae	KT722705	17,154	[64]
*L. viridis viridis*	Lacertidae	Lacertinae	AM176577	17,156	[21]
*Phoenicolacerta*					
*Ph. kulzeri*	Lacertidae	Lacertinae	FJ460596	17,199	[22]
*Podarcis*					
*Po. muralis*	Lacertidae	Lacertinae	FJ460597	17,311	[22]
*Po. siculus*	Lacertidae	Lacertinae	FJ460598	17,297	[22]
*Takydromus*					
*T. amurensis*	Lacertidae	Lacertinae	KU641018	17,333	[65]
*T. kuehnei*	Lacertidae	Lacertinae	MZ435950	17,224	[66]
*T. septentrionalis*	Lacertidae	Lacertinae	MK630237	18,304	[67]
*T. sexlineatus*	Lacertidae	Lacertinae	KF425529	18,943	[68]
*T. sylvaticus*	Lacertidae	Lacertinae	JX290083	17,838	[69]
*T. tachydromoides*	Lacertidae	Lacertinae	AB080237	18,245	[70]
*T. wolteri*	Lacertidae	Lacertinae	JX181764	18,236	[71]
*Zootoca*					
*Z. vivipara*	Lacertidae	Lacertinae	KM401599	17,046	[72]
*Psammodromus*					
*Ps. algirus*	Lacertidae	Gallotiinae	MW496117	17,118	[15]
Outgroup					
*Amphisbaena*					
*Am. schmidti*	Amphisbaenidae	−	AY605475	17,423	[73]
*Bipes*					
*B. biporus*	Bipedidae	−	AY605481	16,430	[73]
*Loxopholis*					
*Lo. percainatum*	Gymnophthalmidae	−	MW864329	15,875	[74]

**Table 3:** Type of conserved structure element of TAS1 in Lacertidae.

Type	Number of Species	TAS1																							
	*L. dugesii*	A	C	T	A	T	T	A	T	G	T	A	T	A	T	A	G	T	G	C	A	T	T	A	A
1	27	·	·	·	·	·	·	·	·	·	·	·	·	·	·	·	·	·	·	·	·	·	·	·	·
2	11	·	·	·	·	·	·	·	·	·	·	·	·	·	·	·	·	·	A	·	·	·	·	·	·
3	1	·	·	·	·	·	·	·	C	·	G	·	·	·	·	·	·	C	·	·	·	·	·	·	C
4	1	·	·	·	·	·	·	·	·	·	·	·	·	·	·	T	·	·	A	·	·	·	·	·	·
5	1	·	·	·	·	·	·	·	·	·	·	·	·	·	·	C	·	·	A	·	·	·	·	·	·
6	1	·	·	A	T	·	·	·	·	·	·	·	·	·	A	T	·	·	A	·	·	·	·	·	·
7	1	·	·	A	·	·	·	·	·	·	·	·	·	·	·	·	T	·	·	·	·	·	·	C	A
8	1	·	·	A	T	·	·	·	·	·	·	·	·	·	·	·	·	·	·	·	·	·	·	·	G
9	1	·	·	·	·	C	·	·	·	·	·	·	·	·	·	·	·	·	·	·	·	·	·	·	·

Types of TAS1 of the control region in Lacertidae. Type 1: *Al. nigropunctatus*, *D. armeniaca*; *D. brauneri*, *D. chlorogaster*, *D. daghestanica*, *D. dahli*, *D. derjugini*, *D. mixta*, *D. parvula*, *D. portschinskii*, *D. raddei*, *D. rudis*, *D. saxicola*, *D. unisexualis*, *D. valentini*, *E. nikolskii*, *E. przewalskii*, *L. agilis*, *L. bilineata*, *L. viridis viridis*, *Ph. kulzeri*, *Po. muralis*, *Po. siculus*, *T. kuehnei*, *T. septentrionalis*, *T. sylvaticus*, *T. wolteri*; Type 2: *Ac. aureus*, *Ac. boskianus*, *Ac. erythrurus*, *Ac. guineensis*, *Ac. schmidti*, *Au. australis*, *E. scripta*, *E. multiocellata*, *E. stummeri*, *E. vermiculata*, *E. yarkandensis*; Type 3: *E. argus*; Type 4: *E. brenchleyi*; Type 5: *E. szczerbaki*; Type 6: *Mes. olivieri*; Type 7: *Pe. laticeps*; Type 8: *Ps. algirus*; Type 9: *Z. vivipara*.

**Table 4:** Type of conserved structure element of CSB1 in Lacertidae.

Type	Number of Species	CSB1																																					
	*L. dugesii*	C	T	A	T	A	T	G	G	T	A	T	T	A	T	T	G	T	C	T	T	A	A	T	G	C	T	T	G	G	T	A	G	A	C	A	T	A	T
1	12	·	·	·	·	·	·	·	·	·	·	·	·	·	·	·	·	·	·	·	·	·	·	·	·	·	·	·	·	·	·	·	·	·	·	·	·	·	·
2	13	·	·	·	·	·	·	·	·	·	·	·	·	·	·	·	C	·	T	·	·	·	·	·	·	·	·	·	·	·	·	·	·	·	·	·	·	·	·
3	2	·	·	·	·	·	·	·	·	·	·	·	·	·	·	·	T	C	·	·	G	·	·	·	·	·	·	·	·	·	·	·	·	·	·	·	·	·	·
4	1	·	·	·	·	·	·	·	·	·	·	·	·	·	·	·	T	C	·	·	·	·	·	·	·	·	·	·	·	·	·	·	·	·	·	·	·	·	·
5	1	·	·	·	·	·	·	·	·	·	·	·	·	·	·	·	T	C	T	·	G	·	·	·	·	·	·	·	·	C	·	·	·	·	·	·	·	·	·
6	1	·	·	·	·	T	·	·	·	·	·	·	·	·	C	·	T	C	A	·	·	·	·	·	·	·	·	·	·	·	·	·	·	·	·	·	·	·	·
7	1	·	·	·	·	·	·	·	·	·	·	·	·	G	·	·	C	·	T	·	·	·	·	·	·	·	·	·	·	·	·	·	·	·	·	·	·	·	·
8	1	·	·	·	·	·	·	·	·	·	G	·	·	·	·	·	·	C	·	·	G	·	·	·	·	·	·	·	·	·	·	·	·	·	·	·	·	·	·
9	2	·	·	·	·	·	·	·	·	·	·	·	·	·	·	·	·	G	A	·	·	·	·	·	·	·	·	·	·	·	·	·	·	·	·	·	·	·	·
10	2	·	·	·	·	·	·	·	·	·	·	·	·	G	·	·	·	·	·	·	·	·	·	·	·	·	·	·	·	·	·	·	·	·	·	·	·	·	·
11	2	·	·	·	·	·	·	·	·	·	·	·	·	·	·	·	·	·	·	·	G	·	·	·	·	·	·	·	·	·	·	·	·	·	·	·	·	·	·
12	3	·	·	·	·	T	·	·	·	·	·	·	·	·	·	·	·	·	·	·	G	·	·	·	·	·	·	·	·	·	·	·	·	·	·	·	·	·	·
13	1	·	·	·	·	·	·	·	·	·	G	·	·	·	·	·	·	A	·	·	G	·	·	·	·	·	·	·	·	·	·	·	·	·	·	·	·	·	·
14	1	·	·	·	·	·	·	·	·	G	G	C	·	·	·	·	·	·	·	G	·	·	·	·	·	·	·	·	·	·	·	·	·	·	·	·	·	·	·
15	1	·	·	·	·	T	·	·	·	·	G	·	·	·	·	·	·	·	·	·	G	·	·	·	·	·	·	·	·	·	·	·	·	·	·	·	·	·	·
16	1	·	·	·	·	T	·	·	·	·	·	·	·	·	·	·	T	A	·	·	G	·	·	·	·	·	·	·	·	·	·	·	·	·	·	·	·	·	·
17	1	·	·	·	·	·	A	·	·	·	·	·	·	·	·	·	·	·	·	·	·	·	·	·	·	·	·	·	·	·	·	·	·	·	·	·	·	·	A
18	1	·	·	·	·	·	·	·	·	·	·	·	·	·	·	·	·	·	A	·	·	·	·	·	·	·	·	·	·	A	·	·	·	·	·	·	·	·	·

Types of CSB1 of the control region in Lacertidae. Type 1: *L. agilis*, *L. bilineata*, *L. viridis viridis*, *Ph. kulzeri*, *Po. muralis*, *Ps. algirus*, *T. kuehnei*, *T. amurensis*, *T. septentrionalis*, *T. sylvaticus*, *T. wolteri*, *Z. vivipara*; Type 2: *D. armeniaca*, *D. caucasica*, *D. chlorogaster*, *D. daghestanica*, *D. dahli*, *D. derjugini*, *D. mixta*, *D. parvula*, *D. portschinskii*, *D. raddei*, *D. rudis*, *D. unisexualis*, *D. valentini*; Type 3: *Mer. squamulosus*, *Pe. laticeps*; Type 4: *Po. siculus*; Type 5: *Mes. olivieri*; Type 6: *E. stummeri*; Type 7: *D. praticola*; Type 8: *E. vermiculata*; Type 9: *Ac. aureus*, *Ac. boskianus*; Type 10: *D. brauneri*, *D. saxicola*; Type 11: *E. scripta*, *E. nikolskii*; Type 12: *E. dzungarica*, *E. przewalskii*, *E. yarkandensis*; Type 13: *E. argus*; Type 14: *E. brenchleyi*; Type 15: *E. multiocellata*; Type 16: *E. szczerbaki*; Type 17: *Ac. guineensis*; Type 18: *Ac. schmidti*.

**Table 5:** Type of conserved structure element of CSB2 in Lacertidae.

Type	Number of Species	CSB2																	
	*L. dugesii*	C	A	A	A	C	C	C	C	C	C	T	A	C	C	C	C	C	C
1	41	·	·	·	·	·	·	·	·	·	·	·	·	·	·	·	·	·	·
2	1	·	·	·	·	T	·	·	·	·	·	·	·	·	·	·	·	·	·
3	1	·	·	·	·	·	·	·	·	·	·	·	·	·	·	T	·	·	·
4	1	T	·	·	·	·	T	·	·	T	·	·	·	·	·	·	·	·	T
5	1	·	·	·	·	T	·	T	·	T	·	·	·	·	·	T	·	·	·
6	1	·	·	·	·	·	T	·	·	·	·	·	G	·	·	·	·	·	T

Types of CSB2 of the control region in Lacertidae. Type 1: *Ac. aureus*, *Ac. boskianus*, *Ac. schmidti*, *Au. australis*, *D. armeniaca*, *D. brauneri*, *D. chlorogaster*, *D. daghestanica*, *D. dahli*, *D. derjugini*, *D. mixta*, *D. parvula*, *D. portschinskii*, *D. praticola*, *D. raddei*, *D. saxicola*, *D. unisexualis*, *D. valentini*, *E. dzungarica*, *E. scripta*, *E. multiocellata*, *E. przewalskii*, *E. stummeri*, *E. szczerbaki*, *E. yarkandensis*, *L. agilis*, *L. bilineata*, *L. viridis viridis*, *Mer. squamulosus*, *Mes. olivieri*, *Pe. laticeps*, *Ph. kulzeri*, *Po. muralis*, *Po. siculus*, *Ps. algirus*, *T. kuehnei*, *T. amurensis*, *T. septentrionalis*, *T. sylvaticus*, *T. wolteri*, *Z. vivipara*; *Type 2: E. nikolskii*; Type 3: *D. rudis*; Type 4: *Ac. erythrurus*; Type 5: *E. argus*; Type 6: *E. brenchleyi*.

**Table 6:** Type of conserved structure element of TAS2 in Lacertidae.

Type	Number of Species	TAS2									
	*L. dugesii*	C	A	T	A	C	A	T	T	A	A
1	1	·	·	·	·	·	·	·	·	·	·
2	13	T	·	·	G	·	·	·	·	·	·
3	5	T	·	·	·	·	·	·	·	·	·
4	5	T	·	·	·	·	·	·	·	·	T
5	2	T	·	·	·	T	·	·	·	·	·
6	1	T	·	·	·	A	·	·	·	·	·
7	1	T	·	·	·	·	·	·	·	·	C
8	1	T	·	·	·	·	·	·	A	·	T
9	1	T	·	·	C	·	·	·	·	·	·
10	3	A	·	·	·	·	·	·	·	·	T
11	1	A	G	·	·	·	·	·	·	·	·
12	1	A	·	·	·	·	·	·	·	·	·
13	1	·	·	·	·	·	·	·	·	·	T
14	2	·	·	·	G	·	·	·	·	·	·
15	1	·	·	·	G	T	·	·	·	·	·
16	1	·	G	·	·	·	·	·	·	·	·
17	1	·	·	·	·	T	·	·	·	T	·
18	2	·	·	·	·	T	·	·	·	·	·
19	1	·	·	·	·	·	T	·	·	·	·
20	1	·	·	·	·	T	·	·	T	·	·
21	1	·	·	·	·	A	C	·	·	G	·
22	1	·	·	·	C	·	G	A	·	·	·
23	1	·	·	·	A	·	·	T	·	·	·
24	1	·	·	·	·	·	·	·	A	·	T
25	1	·	·	·	·	·	·	·	·	C	·
26	1	·	·	·	·	·	A	·	·	·	·

Types of TAS2 of the control region in Lacertidae. Type 1: *L. viridis viridis*; Type 2: *D. armeniaca*, *D. brauneri*, *D. daghestanica*, *D. dahli*, *D. mixta*, *D. parvula*, *D. portschinskii*, *D. rudis*, *D. saxicola*, *D. valentini*, *E. przewalskii*, *L. agilis*, *Po. siculus*; Type 3: *D. derjugini*, *D. raddei*, *E. yarkandensis*, *Ph. kulzeri*, *L. bilineata*; Type 4: *Ac. schmidti*, *E. dzungarica*, *E. nikolskii*, *E. szczerbaki*, *Po. muralis*; Type 5: *D. chlorogaster*, *D. clarkorum*; Type 6: *T. amurensis*; Type 7: *D. unisexualis*; Type 8: *Ac. erythrurus*; Type 9: *D. caucasica*; Type 10: *Ac. boskianus*, *E. multiocellata*, *E. stummeri*; Type 11: *Ac. guineensis*; Type 12: *E. vermiculata*; Type 13: *Ac. aureus*; Type 14: *Au. australis*, *Pe. laticeps*; Type 15: *Mes. olivieri*; Type 16: *Z. vivipara*; Type 17: *Al. nigropunctatus*; Type 18: *T. septentrionalis*, *T. wolteri*; Type 19: *T. sexlineatus*; Type 20: *T. sylvaticus*; Type 21: *D. praticola*; Type 22: *E. argus*; Type 23: *E. brenchleyi*; Type 24: *E. scripta*; Type 25: *Ps. algirus*; Type 26: *T. kuehnei*.

**Table 7:** Type of conserved structure element of CSB3 in Lacertidae.

Type	Number of Species	CSB3																			
	*L. dugesii*	T	C	G	C	C	A	A	A	C	C	C	C	T	A	A	A	A	C	G	A
1	36	·	·	·	·	·	·	·	·	·	·	·	·	·	·	·	·	·	·	·	·
2	5	·	·	·	·	·	·	·	·	·	·	·	·	A	·	·	·	·	·	·	·
3	1	·	·	·	T	·	·	·	·	·	·	A	·	·	·	·	·	·	·	·	·
4	1	·	·	·	·	·	·	·	·	T	·	·	·	·	·	·	·	·	·	·	·
5	1	C	·	·	·	·	·	·	·	T	T	·	·	C	G	·	·	·	·	C	·
6	1	·	·	·	·	·	·	·	·	·	·	T	·	G	·	·	·	·	·	A	·
7	1	·	·	·	·	·	·	·	·	T	·	·	·	·	·	G	·	·	·	A	G
8	1	A	G	G	·	·	·	A	T	T	·	·	·	·	·	·	T	·	T	·	·

Types of CSB3 of the control region in Lacertidae. Type 1: *Au. australis*, *D. brauneri*, *D. chlorogaster*, *D. clarkorum*, *D. daghestanica*, *D. dahli*, *D. derjugini*, *D. mixta*, *D. parvula*, *D. portschinskii*, *D. praticola*, *D. raddei*, *D. rudis*, *D. saxicola*, *D. unisexualis*, *D. valentini*, *E. dzungarica*, *E. scripta* KZL15, *E. scripta* KZL44, *E. multiocellata*, *E. przewalskii*, *E. stummeri*, *E. szczerbaki*, *E. yarkandensis*, *L. agilis*, *L. bilineata*, *L. viridis viridis*, *Ph. kulzeri*, *Po. muralis*, *Po. siculus*, *T. amurensis*, *T. kuehnei*, *T. septentrionalis*, *T. sylvaticus*, *T. wolteri*, *Z. vivipara*; Type 2: *Ac. aureus*, *Ac. boskianus*, *Ac. schmidti*, *Mer. squamulosus*, *Pe. laticeps*; Type 3: *D. armeniaca*; Type 4: *E. nikolskii*; Type 5: *E. argus*; Type 6: *E. brenchleyi*; Type 7: *E. vermiculata*; Type 8: *Ps. algirus*.

## Data Availability

Illumina raw reads are deposited at NCBI Sequence Read Archive (SRA) under accession nos. SRR16509497 for *E. nikolskii* in the BioProject PRJNA773219, SRR16508134 for *E. yarkandensis* in the BioProject PRJNA773190, PRJNA779878 for *E. szczerbaki* in the BioProject PRJNA779878, SRR18381820 for *E. scripta* KZL15 in the BioProject PRJNA817793, and SRR18441150 for *E. scripta* KZL44 in the BioProject PRJNA817796. The mitochondrial genomes are deposited at GenBank with accession nos. OK587334, OK585048, OL457296, OM935765, and OM935766, respectively.

## Supplementary Materials

The following supporting information can be downloaded at: https://www.mdpi.com/article/10.3390/genes13050726/s1. Figure S1: Putative secondary structures of the tRNAs identified in the mitogenomes of (a), *E. scripta* KZL15; (b), *E. scripta* KZL44; (c), *E. nikolskii*; (d), *E. szczerbaki*; (e), *E. yarkandensis*. Figure S2: Maximum likelihood tree inferred from 13 concatenated PCGs. Node numbers represent ultrafast bootstrap support. GenBank accession numbers are given with species names, and subfamily assignments are listed, along with viviparous group/subgenera in *Eremias*. Table S1: Best-fit models and partitioning schemes selected using PartitionFinder 2. Table S2: Organization of five mitochondrial genomes of *Eremias* determined in this study. (a), *E. scripta* KZL15; (b), *E. scripta* KZL44; (c), *E. nikolskii*; (d), *E. szczerbaki*; (e), *E. yarkandensis*. Table S3: The A+T composition of the complete mitogenomes, PCGs, rRNAs, tRNAs and CR, and composition skew values in Lacertidae. Table S4: Position of conserved structural elements of the CR in Lacertidae. Table S5: Compositions of conserved structures of the CR in Lacertidae. Table S6: Tandem repeats of the CR in Lacertidae. Table S7: Uncorrected genetic distances (p-distances) within and between the subgenus *Pareremias* and *E. nikolskii* based on 13 concatenated PCGs.

## References

[1] Anderson S., Bankier A.T., Barrell B.G., de Bruijn M.H.L., Coulson A.R., Drouin J.I., Eperon C., Nierlich D.P., Roe B.A., Sanger F. (1981). Sequence and organization of the human mitochondrial genome. Nature.

[2] Boore J.L. (1999). Animal mitochondrial genomes. Nucleic Acids Res..

[3] Wolstenholme D.R. (1992). Animal mitochondrial DNA: Structure and evolution. Int. Rev. Cytol..

[4] Brehm A., Harris D.J., Alves C.D., Jesus J.D., Thomarat F.D., Vicente L.D. (2003). Structure and evolution of the mitochondrial DNA complete control region in the lizard *Lacerta dugesii* (Lacertidae, Sauria). J. Mol. Evol..

[5] Hoelzel A.R., Lopez J.V., Dover G.A., O’Brien S.J. (1994). Rapid evolution of a heteroplasmic repetitive sequence in the mitochondrial DNA control region of carnivores. J. Mol. Evol..

[6] Wu N., Liu J., Wang S., Guo X. (2022). Comparative analysis of mitochondrial genomes in two subspecies of the sunwatcher toad-headed agama (*Phrynocephalus helioscopus*): Prevalent intraspecific gene rearrangements in *Phrynocephalus*. Genes.

[7] Sbisà E., Tanzariello F., Reyes A., Pesole G., Saccone C. (1997). Mammalian mitochondrial D-loop region structural analysis: Identification of new conserved sequences and their functional and evolutionary implications. Gene.

[8] Harris D.J., Arnold E.N., Thomas R.H. (1998). Relationships of lacertid lizards (Reptilia: Lacertidae) estimated from mitochondrial DNA sequences and morphology. Proc. Biol. Sci. B.

[9] Mayer W., Pavlicev M. (2007). The phylogeny of the family Lacertidae (Reptilia) based on nuclear DNA sequences: Convergent adaptations to arid habitats within the subfamily Eremiainae. Mol. Biol. Evol..

[10] Pavlicev M., Mayer W. (2009). Fast radiation of the subfamily Lacertinae (Reptilia: Lacertidae): History or methodical artefact?. Mol. Phylogenet. Evol..

[11] Uetz P., Freed P., Aguilar R., Hošek J. The Reptile Database. http://www.reptile-database.org.

[12] Arnold E.N., Arribas O., Carranza S. (2007). Systematics of the Palaearctic and Oriental lizard tribe Lacertini (Squamata: Lacertidae: Lacertinae), with descriptions of eight new genera. Zootaxa.

[13] Hipsley C.A., Himmelmann L., Metzler D., Müller J. (2009). Integration of Bayesian molecular clock methods and fossil-based soft bounds reveals early Cenozoic origin of African lacertid lizards. BMC Evol. Biol..

[14] Mendes J., Harris D.J., Carranza S., Salvi D. (2016). Evaluating the phylogenetic signal limit from mitogenomes, slow evolving nuclear genes, and the concatenation approach. New insights into the Lacertini radiation using fast evolving nuclear genes and species trees. Mol. Phylogenet. Evol..

[15] Kirchhof S., Lyra M.L., Rodríguez A., Ineich I., Müller J., Rödel M.O., Trape J.F., Vences M., Boissinot M. (2021). Mitogenome analyses elucidate the evolutionary relationships of a probable Eocene wet tropics relic in the xerophilic lizard genus *Acanthodactylus*. Sci. Rep..

[16] Guo X., Dai X., Dali C., Papenfuss T.J., Ananjeva N.B., Melnikov D.A., Wang Y. (2011). Phylogeny and divergence times of some racerunner lizards (Lacertidae: *Eremias*) inferred from mitochondrial 16S rRNA gene segments. Mol. Phylogenet. Evol..

[17] Khan M.A., Jablonski D., Nadeem M.S., Masoor R., Kehlmaier C., Spitzweg C., Fritz U. (2021). Molecular phylogeny of *Eremias* spp. from Pakistan contributes to a better understanding of the diversity of racerunners. J. Zool. Syst. Evol. Res..

[18] Liu J.L., Dujsebayeva T.N., Chirikova M.A., Gong X., Li D.J., Guo X.G. (2021). Does the Dzungarian racerunner (*Eremias dzungarica* Orlova, Poyarkov, Chirikova, Nazarov, Munkhbaatar, Munkhbayar &Terbish, 2017) occur in China? Species delimitation and identification with DNA barcoding and morphometric analyses. Zool. Res..

[19] Wang S., Liu J.L., Zhang B., Guo X.G. (2021). The complete mitochondrial genome of *Eremias dzungarica* (Reptilia, Squamata, Lacertidae) from the Junggar Basin in Northwest China. Mitochondrial DNA B.

[20] Brown R.P. (2005). Large subunit mitochondrial rRNA secondary structures and site-specific rate variation in two lizard lineages. J. Mol. Evol..

[21] Böhme M.U., Fritzsch G., Tippmann A., Schlegel M., Berendonk T.U. (2007). The complete mitochondrial genome of the green lizard *Lacerta viridis viridis* (Reptilia: Lacertidae) and its phylogenetic position within squamate reptiles. Gene.

[22] Podnar M., Pinsker W., Mayer W. (2009). Complete mitochondrial genomes of three lizard species and the systematic position of the Lacertidae (Squamata). J. Zool. Syst. Evol. Res..

[23] Jauss R.-T., Solf N., Kolora S.R.R., Schaffer S., Wolf R., Henle K., Fritz U., Schlegel M. (2021). Mitogenome evolution in the *Lacerta viridi*s complex (Lacertidae, Squamata) reveals phylogeny of diverging clades. Syst. Biodivers..

[24] Stoneking M., Hedgecock D., Higuchi R.G., Vigilant L., Erlich H.A. (1991). Population variation of human mtDNA control region sequences detected by enzymatic amplification and sequence-specific oligonucleotide probes. Am. J. Hum. Genet..

[25] Aquadro C.F., Greenberg B.D. (1983). Human mitochondrial DNA variation and evolution: Analysis of nucleotide sequences from seven individuals. Genetics.

[26] Kasamatsu H., Robberson D.L., Vinograd J. (1971). A novel closed-circular mitochondrial DNA with properties of a replicating intermediate. Proc. Natl. Acad. Sci. USA.

[27] Lee W.J., Conroy J., Howell W.H., Kocher T.D. (1995). Structure and evolution of teleost mitochondrial control regions. J. Mol. Evol..

[28] Randi E., Lucchini V. (1998). Organization and evolution of the mitochondrial DNA control region in the avian genus Alectoris. Mol. Evol..

[29] Liu H., Yang G., Wei F.W., Li M., Hu J.C. (2003). Sequence variability of the mitochondrial DNA control region and population genetic structure of sika deers (*Cervus nippon*) in China. Acta Zool. Sinica.

[30] Matson C.W., Baker R.J. (2001). DNA sequence variation in the mitochondrial control region of red-backed voles (*Clethrionomys*). Mol. Biol. Evol..

[31] Chen M., Liu J., Chen D., Guo X. (2020). Complete mitochondrial genome of a blue-tailed skink *Plestiodon capito* (Reptilia, Squamata, Scincidae) and comparison with other Scincidae lizards. Genetica.

[32] Aljanabi S.M., Martinez I. (1997). Universal and rapid salt-extraction of high quality genomic DNA for PCR-based techniques. Nucleic Acids Res..

[33] Chen S., Zhou Y., Chen Y., Gu J. (2018). fastp: An ultra-fast all-in-one fastq preprocessor. Bioinformatics.

[34] Fastp. https://github.com/OpenGene/fastp.

[35] Bankevich A., Nurk S., Antipov D., Gurevich A.A., Dvorkin M., Kulikov A.S., Lesin V.M., Nikolenko S.I., Pham S., Prjibelski A.D. (2012). SPAdes: A new genome assembly algorithm and its applications to single-cell sequencing. J. Comput. Biol..

[36] SPAdes-v3.10.1. http://cab.spbu.ru/software/spades/.

[37] Web BankIt. http://www.ncbi.nlm.nih.go-v/BankIt/index.html.

[38] Web BLAST. https://blast.ncbi.nlm.nih.gov/Blast.cgi.

[39] Bernt M., Donath A., Jühling F., Externbrink F., Florentz C., Fritzsch G., Pütz J., Middendorf M., Stadler P.F. (2013). MITOS: Improved de novo metazoan mitochondrial genome annotation. Mol. Phylogenet. Evol..

[40] MITOS Web Server. http://mitos.bioinf.uni-leipzig.de/index.py.

[41] tRNAscan-SE Web Server. http://lowelab.ucsc.edu/tRNAscan-SE/.

[42] Lowe T.M., Chan P.P. (2016). tRNAscan-SE On-line integrating search and context for analysis of transfer RNA genes. Nucleic Acids Res..

[43] Web RNAfold. http://rna.tbi.univie.ac.at//cgi-bin/RNAWebSuite/RNAfold.cgi.

[44] Comparative RNA Web (CRW) Site. http://www.rna.ccbb.utexas.edu/.

[45] Hickson R.E., Simon C., Cooper A., Spicer G.S., Sullivan J., Penny D. (1996). Conserved sequence motifs, alignment, and secondary structure for the third domain of animal 12S rRNA. Mol. Biol. Evol..

[46] Cannone J.J., Subramanian S., Schnare M.N., Collett J.R., D’Souza L.M., Du Y., Feng B., Lin N., Madabusi L.V., Müller K.M. (2002). The Comparative RNA Web (CRW) Site: An online database of comparative sequence and structure information for ribosomal, intron, and other RNAs. BMC Bioinform..

[47] Greiner S., Lehwark P., Bock R. (2019). OrganellarGenomeDRAW (OGDRAW) version 1.3.1: Expanded toolkit for the graphical visualization of organellar genomes. Nucleic Acids Res..

[48] OGDRAW. https://chlorobox.mpimp-golm.mpg.de/OGDraw.html.

[49] Kumar S., Stecher G., Tamura K. (2016). MEGA7: Molecular evolutionary genetics analysis version 7.0 for bigger datasets. Mol. Biol. Evol..

[50] Benson G. (1999). Tandem repeats finder: A program to analyze DNA sequences. Nucleic Acids Res..

[51] Tandem Repeats Finder Web Server. http://tandem.bu.edu/trf/trf.html.

[52] Du Y., Qiu Q.B., Tong Q.L., Lin L.H. (2016). The complete mitochondrial genome of *Eremias przewalskii* (Squamata: Lacertidae). Mitochondrial DNA A.

[53] Zhou T., Li D., Dujsebayeva T.N., Liu J., Guo X. (2016). Complete mitochondrial genome of Stummer’s Racerunner (*Eremias stummeri*) from Kazakhstan. Mitochondrial DNA A.

[54] Tong Q.L., Yao Y.T., Lin L.H., Ji X. (2016). The complete mitochondrial genome of *Eremias vermiculata* (Squamata: Lacertidae). Mitochondrial DNA.

[55] Rui J.L., Wang Y.T., Nie L.W. (2009). The complete mitochondrial DNA genome of *Eremias brenchleyi* (Reptilia: Lacertidae) and its phylogeny position within Squamata reptiles. Amphib-Reptil..

[56] Li D., Song S. (2015). Complete mitochondrial genome of *Eremias multiocellata*.

[57] Su X., Liu J., Chen D., Guo X. (2019). Next-generation sequencing yields a nearly complete mitochondrial genome of the Multiocellated Racerunner (*Eremias multiocellata*) in Northwest China. Mitochondrial DNA B.

[58] Kim S.K., Yoo D.U., Hwang U.W. (2016). Complete mitochondrial genome of Mongolia racerunner (*Eremias argus*).

[59] Yu X., Lin L. (2021). The partial mitochondrial genome of *Eremias arguta*.

[60] Zhou Z.S., Li H., Tong Q.L., Lin L.H., Ji X. (2016). The nearly complete mitochondrial genome of the rapid racerunner *Eremias velox* (Squamata: Lacertidae). Mitochondrial DNA A.

[61] Murtskhvaladze M., Tarkhnishvili D., Anderson C.L., Kotorashvili A. (2020). Phylogeny of caucasian rock lizards (*Darevskia*) and other true lizards based on mitogenome analysis: Optimisation of the algorithms and gene selection. PLoS ONE.

[62] Tao C.R. (2014). The Complete Mitogenome of *Lacerta agilis* and the Phylogenetic Analysis of Squamata. Master’s Dissertation.

[63] Margaryan A. (2020). The partial mitochondrial genome of *Lacerta agilis*.

[64] Kolora S.R., Faria R., Weigert A., Schaffer S., Grimm A., Henle K., Sahyoun A.H., Stadler P.F., Nowick K., Bleidorn C. (2017). The complete mitochondrial genome of *Lacerta bilineata* and comparison with its closely related congener *L. viridis*. Mitochondrial DNA A.

[65] Ma W.W., Liu H., Zhao W.G., Liu P. (2016). The complete mitochondrial genome of *Takydromus amurensis* (Squamata: Lacertidae). Mitochondrial DNA B.

[66] Wu L.X., Luo K.N., Ding G.H. (2021). Complete mitochondrial genome of *Takydromus kuehnei*.

[67] Hu J.G., Peng L.F., Tang X.S., Huang S. (2019). The complete mitochondrial genome of *Takydromus septentrionalis* (Reptilia: Lacertidae). Mitochondrial DNA B.

[68] Qin P.S., Zeng D.L., Hou L.X., Yang X.W., Qin X.M. (2015). Complete mitochondrial genome of *Takydromus sexlineatus* (Squamata, Lacertidae). Mitochondrial DNA.

[69] Tang X.S., Chen J.M., Huang S. (2014). Mitochondrial genome of the Chung-an ground lizard *Takydromus sylvaticus* (Reptilia: Lacertidae). Mitochondrial DNA.

[70] Kumazawa Y. (2007). Mitochondrial genomes from major lizard families suggest their phylogenetic relationships and ancient radiations. Gene.

[71] Yu D.N., Ji X. (2013). The complete mitochondrial genome of *Takydromus wolteri* (Squamata: Lacertidae). Mitochondrial DNA.

[72] Liu P., Zhu D., Zhao W.G., Ji X. (2016). The complete mitochondrial genome of the common lizard *Zootoca vivipara* (Squamata: Lacertidae). Mitochondrial DNA.

[73] Macey J.R., Papenfuss T.J., Kuehl J.V., Fourcade H.M., Boore J.L. (2004). Phylogenetic relationships among amphisbaenian reptiles based on complete mitochondrial genome sequences. Mol. Phylogenet. Evol..

[74] Brunes T.O., Lyra M.L., Maldonado J.A., Pellegrino K.C.M., Rodrigues M.T., Fujita M.K. (2021). The first mitochondrial genome of a South America parthenogenetic lizard (Squamata: Gymnophthalmidae). Mitochondrial DNA B.

[75] GenBank. https://www.ncbi.nlm.nih.gov/genbank/.

[76] Wiens J.J., Hutter C.R., Mulcahy D.G., Noonan B.P., Townsend T.M., Sites J.W., Reeder T.W. (2012). Resolving the phylogeny of lizards and snakes (Squamata) with extensive sampling of genes and species. Biol. Lett..

[77] Pyron R.A., Burbrink F.T., Wiens J.J. (2013). A phylogeny and revised classification of Squamata, including 4161 species of lizards and snakes. BMC Evol. Biol..

[78] Zheng Y., Wiens J.J. (2016). Combining phylogenomic and supermatrix approaches, and a time-calibrated phylogeny for squamate reptiles (lizards and snakes) based on 52 genes and 4162 species. Mol. Phylogenet. Evol..

[79] Zhang D., Gao F., Li W.X., Jakovlić I., Zou H., Zhang J., Wang G.T. (2020). PhyloSuite: An integrated and scalable desktop platform for streamlined molecular sequence data management and evolutionary phylogenetics studies. Mol. Ecol. Resour..

[80] Lanfear R., Frandsen P.B., Wright A.M., Senfeld T., Calcott B. (2017). PartitionFinder 2: New methods for selecting partitioned models of evolution for molecular and morphological phylogenetic analyses. Mol. Biol. Evol..

[81] Ronquist F., Huelsenbeck J.P. (2003). Mrbayes 3: Bayesian phylogenetic inference under mixed models. Bioinformatics.

[82] Ronquist F., Teslenko M., van der Mark P., Ayres D.L., Darling A., Höhna S., Larget B., Liu L., Suchard M.A., Huelsenbeck J.P. (2012). MrBayes 3.2: Efficient Bayesian phylogenetic inference and model choice across a large model space. Syst. Biol..

[83] Rambaut A., Drummond A.J., Xie D., Baele G., Suchard M.A. (2018). Posterior summarization in Bayesian phylogenetics using Tracer 1.7. Syst. Biol..

[84] Erixon P., Svennblad B., Britton T., Oxelman B. (2003). Reliability of Bayesian posterior probabilities and bootstrap frequencies in phylogenetics. Syst. Biol..

[85] Huelsenbeck J.P., Rannala B. (2004). Frequentist properties of Bayesian posterior probabilities of phylogenetic trees under simple and complex substitution models. Syst. Biol..

[86] Nguyen L.T., Schmidt H.A., von Haeseler A., Minh B.Q. (2015). IQ-TREE: A fast and effective stochastic algorithm for estimating maximum-likelihood phylogenies. Mol. Biol. Evol..

[87] Minh B.Q., Nguyen M.A.T., Haeseler A.V. (2013). Ultrafast approximation for phylogenetic bootstrap. Mol. Biol. Evol..

[88] FigTree. http://tree.bio.ed.ac.uk/software/figtree/.

[89] Yu P., Zhou L., Yang W.T., Miao L.J., Li Z., Zhang X.J., Wang Y., Gui J.F. (2021). Comparative mitogenome analyses uncover mitogenome features and phylogenetic implications of the subfamily Cobitinae. BMC Genomics.

[90] Topal M.D., Fresco J.R. (1976). Complementary base pairing and the origin of substitution mutations. Nature.

[91] Castellana S., Vicario S., Saccone C. (2011). Evolutionary patterns of the mitochondrial genome in Metazoa: Exploring the role of mutation and selection in mitochondrial protein coding genes. Genome Biol. Evol..

[92] Southern S.O., Southern P.J., Dizon A.E. (1988). Molecular characterization of a cloned dolphin mitochondrial genome. J. Mol. Evol..

[93] Brown G.G., Gadaleta G., Pepe G., Saccone C., Sbisà E. (1986). Structural conservation and variation in the D-loop-containing region of vertebrate mitochondrial DNA. J. Mol. Biol..

[94] Orlova V.F., Poyarkov N.A., Chirikova M.A., Nazarov R.A., Munkhbaatar M., Munkhbayar K., Terbish M. (2017). MtDNA differentiation and taxonomy of Central Asian racerunners of *Eremias multiocellata-E. przewalskii* species complex (Squamata, Lacertidae). Zootaxa.

[95] Dixon M.T., Hillis D.M. (1993). Ribosomal RNA secondary structure: Compensatory mutations and implications for phylogenetic analysis. Mol. Biol. Evol..

[96] Springer M.S., Hollar L.J., Burk A. (1995). Compensatory substitutions and the evolution of the mitochondrial 12S rRNA gene in mammals. Mol. Biol. Evol..

[97] Kjer K.M. (1995). Use of rRNA secondary structure in phylogenetic studies to identify homologous positions: An example of alignment and data presentation from the frogs. Mol. Phylogenet. Evol..

[98] Hudelot C., Gowri-Shankar V., Jow H., Rattray M., Higgs P.G. (2003). RNA-based phylogenetic methods: Application to mammalian mitochondrial RNA sequences. Mol. Phylogenet. Evol..

[99] Schöniger M., von Haeseler A. (1994). A stochastic model for the evolution of autocorrelated DNA sequences. Mol. Phylogenet. Evol..

[100] Telford M.J., Wise M.J., Gowri-Shankar V. (2005). Consideration of RNA secondary structure significantly improves likelihood-based estimates of phylogeny: Examples from the Bilateria. Mol. Biol. Evol..

[101] Jow H., Hudelot C., Rattray M., Higgs P.G. (2002). Bayesian phylogenetics using an RNA substitution model applied to early mammalian evolution. Mol. Biol. Evol..

[102] Li J., Wang X., Kong X., Zhao K., He S., Mayden R.L. (2008). Variation patterns of the mitochondrial 16S rRNA gene with secondary structure constraints and their application to phylogeny of cyprinine fishes (Teleostei: Cypriniformes). Mol. Phylogenet. Evol..

[103] Kimura M. (1977). Preponderance of synonymous changes as evidence for the neutral theory of molecular evolution. Nature.

[104] Yang Z., Bielawski J.P. (2000). Statistical methods for detecting molecular adaptation. Trends Ecol. Evol..

[105] Magnus W.J., Rute R.D.F., Louis B., Michael M.H. (2016). Comparative analysis of complete mitochondrial genomes suggests that relaxed purifying selection is driving high nonsynonymous evolutionary rate of the NADH2 gene in whitefish (*Coregonus* ssp.). Mol. Phylogenet. Evol..

[106] Melville R.V. (1985). Opinion 1318 (Opinion correcting the ruling given in Opinion 92) *Lacerta velox* Pallas, 1771 is the type species of *Eremias* Wiegmann, 1834. Bull. Zooll. Nomencl..

[107] Brown W.M., George M., Wilson A.C. (1979). Rapid evolution of animal mitochondrial DNA. Proc. Natl. Acad. Sci. USA.

[108] Doda J.N., Wright C.T., Clayton D.A. (1981). Elongation of displacement loop strands in human and mouse mitochondrial DNA is arrested near specific template sequences. Proc. Natl. Acad. Sci. USA.

[109] Saccone C., Pesole G., Sbisà E. (1991). The main regulatory region of mammalian mitochondrial DNA: Structure-function model and evolutionary pattern. J. Mol. Evol..

[110] Gao S., Tian X., Chang H., Sun Y., Wu Z., Cheng Z., Dong P., Zhao Q., Ruan J., Bu W. (2018). Two novel lncRNAs discovered in human mitochondrial DNA using PacBio full-length transcriptome data. Mitochondrion.

[111] Ji H., Xu X., Jin X., Yin H., Luo J., Liu G., Zhao Q., Chen Z., Bu W., Gao S. (2019). Using high-resolution annotation of insect mitochondrial DNA to decipher tandem repeats in the control region. RNA Biol..

[112] Xu X., Ji H., Jin X., Cheng Z., Yao X., Liu Y., Zhao Q., Zhang T., Ruan J., Bu W. (2019). Using pan RNA-seq analysis to reveal the ubiquitous existence of 5′ and 3′ end small RNAs. Front. Genet..

