# Supplementary material for: Complete Mitochondrial Genomes of Five Racerunners (Lacertidae: Eremias) and Comparison with Other Lacertids: Insights into the Structure and Evolution of the Control Region
Source: Genes (Basel). 2022 Apr 21;13(5):726. doi: 10.3390/genes13050726 (PMC9141765; doi:10.3390/genes13050726)

(a) *Eremias scripta* KZL15

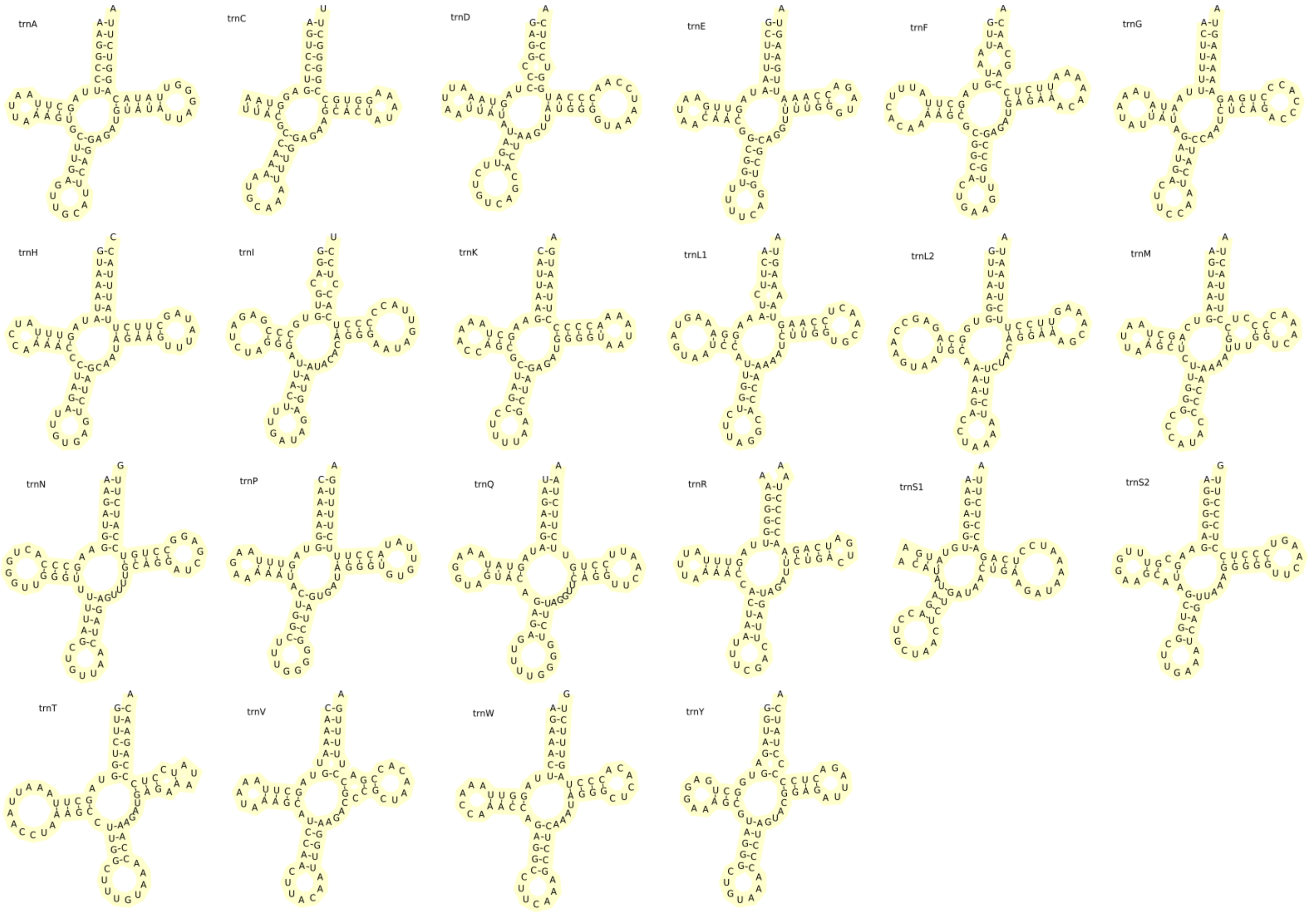

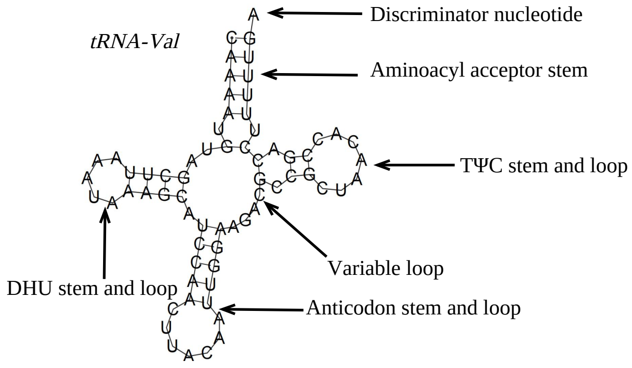

(b) *Eremias scripta* KZL44

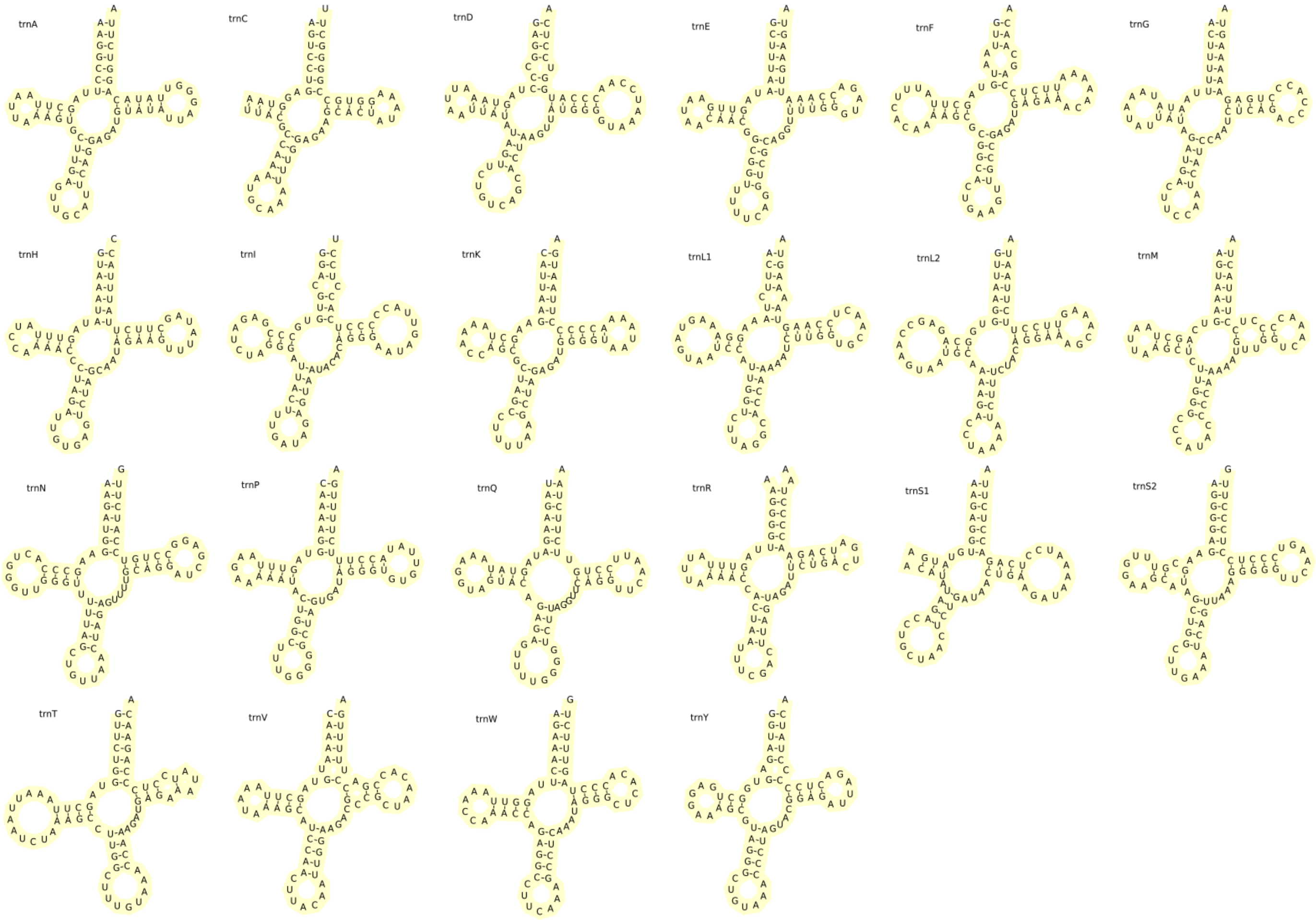

(c) *Eremias nikolskii*

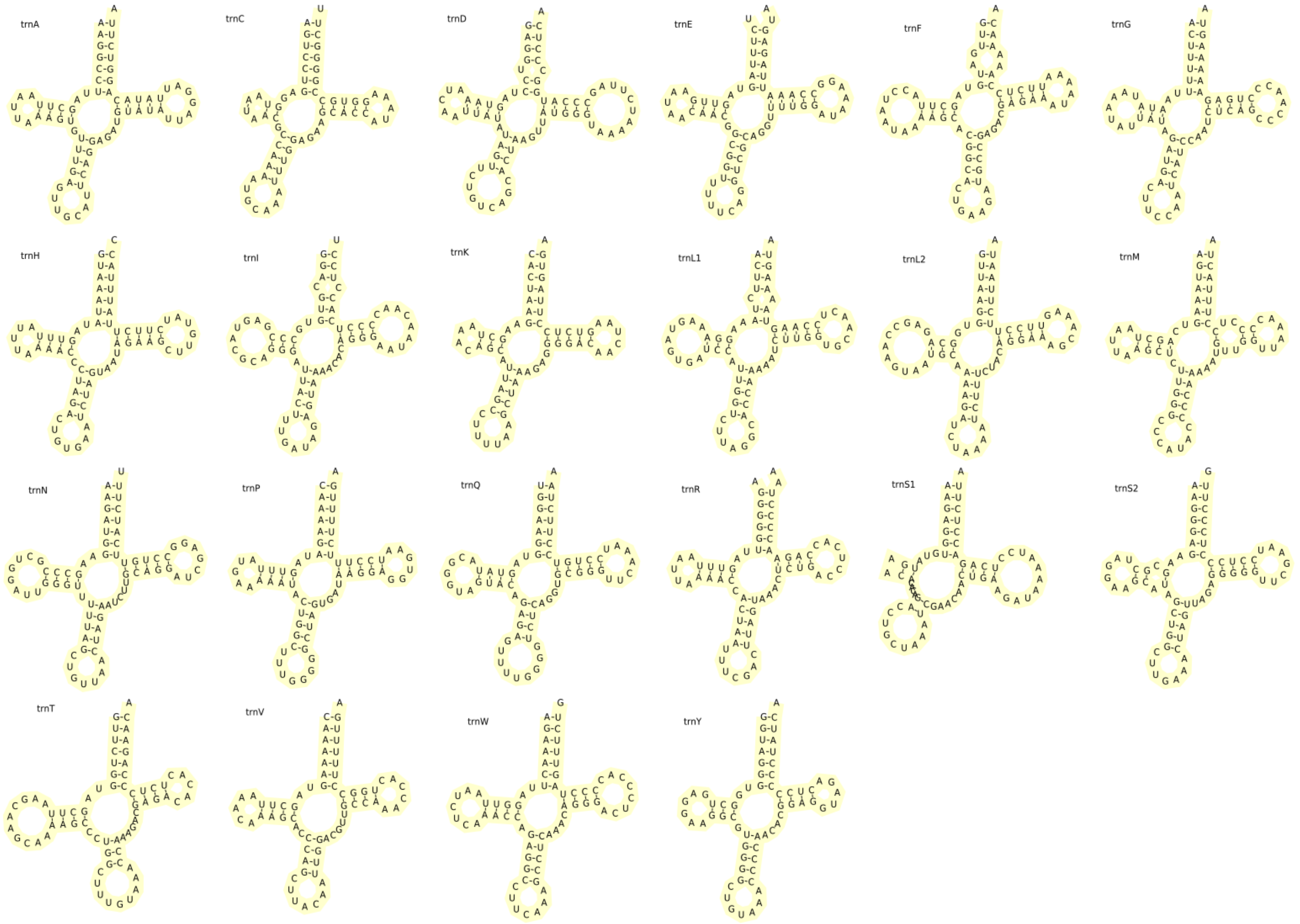

(d) *Eremias szczerbaki*

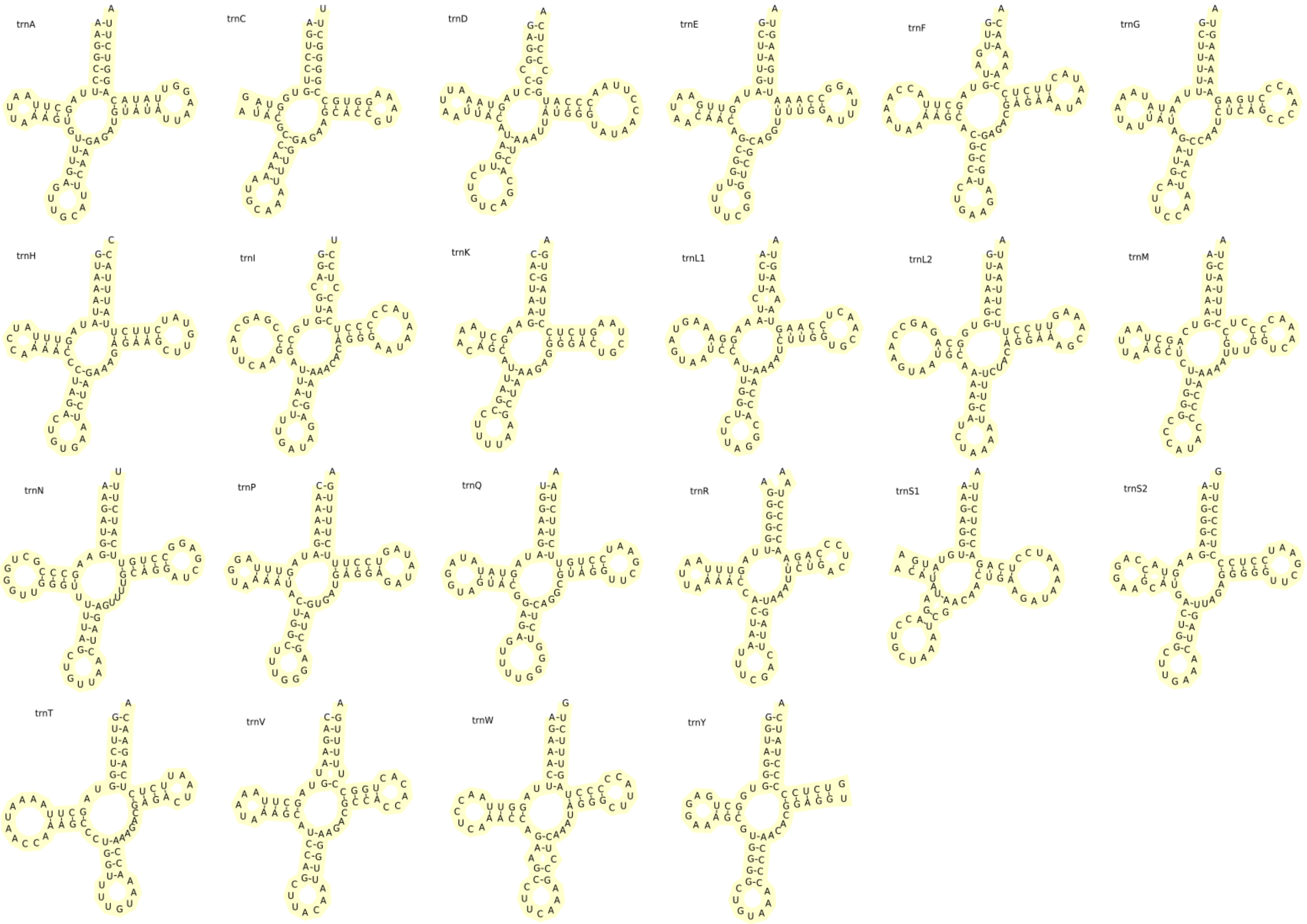

(e) *Eremias yarkandensis*

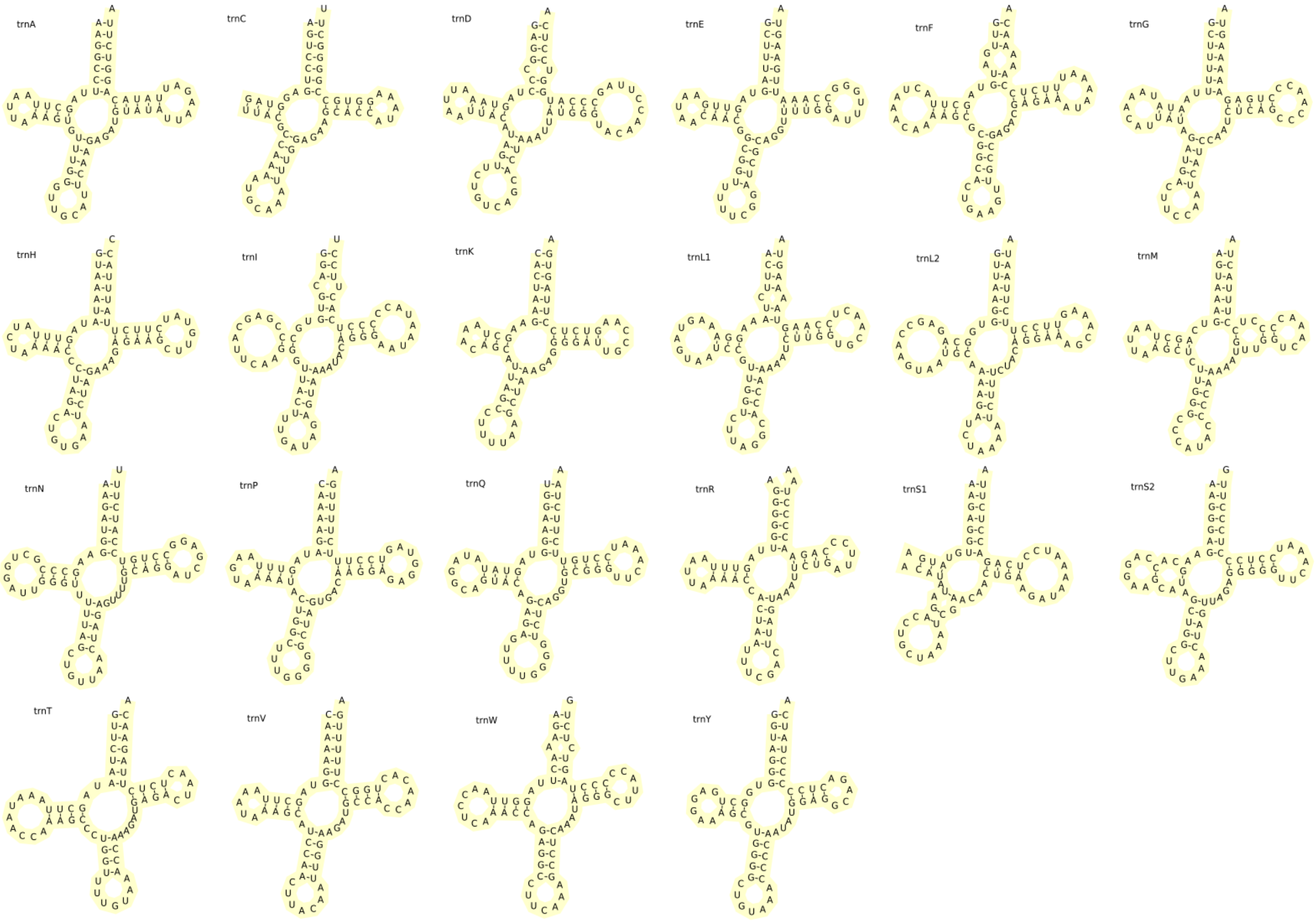

Supplement: Supplementary file 1 [file genes-13-00726-s001.zip › genes-1679660-Figure S1.pdf]
