# Supplementary material for: Complete Mitochondrial Genomes of Five Racerunners (Lacertidae: Eremias) and Comparison with Other Lacertids: Insights into the Structure and Evolution of the Control Region
Source: Genes (Basel). 2022 Apr 21;13(5):726. doi: 10.3390/genes13050726 (PMC9141765; doi:10.3390/genes13050726)
Supplement: Supplementary file 1 [file genes-13-00726-s001.zip › genes-1679660-Figure S2.pdf]

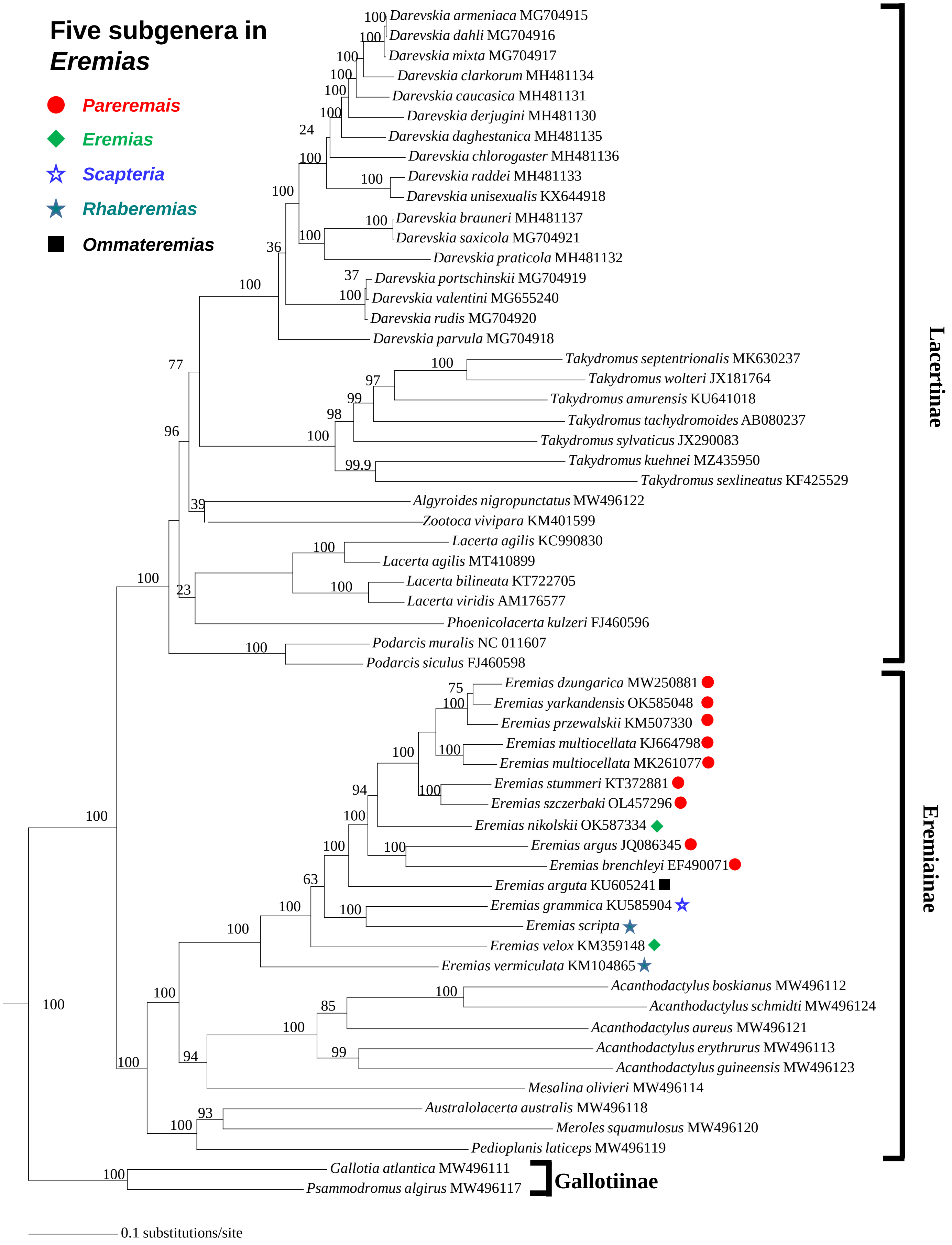

Five subgenera in Eremias
Pareremais
Eremias
Scapteria
Rhaberemias
Ommateremias
Darevskia armeniaca MG704915
Darevskia dahli MG704916
Darevskia mixta MG704917
Darevskia clarkorum MH481134
Darevskia caucasica MH481131
Darevskia derjugini MH481130
Darevskia daghestanica MH481135
Darevskia chlorogaster MH481136
Darevskia raddei MH481133
Darevskia unisexualis KX644918
Darevskia brauneri MH481137
Darevskia saxicola MG704921
Darevskia praticola MH481132
Darevskia portschinskii MG704919
Darevskia valentini MG655240
Darevskia rudis MG704920
Darevskia parvula MG704918
Takydromus septentrionalis MK630237
Takydromus wolteri JX181764
Takydromus amurensis KU641018
Takydromus tachydromoides AB080237
Takydromus sylvaticus JX290083
Takydromus kuehnei MZ435950
Takydromus sexlineatus KF425529
Algyroides nigropunctatus MW496122
Zootoca vivipara KM401599
Lacerta agilis KC990830
Lacerta agilis MT410899
Lacerta bilineata KT722705
Lacerta viridis AM176577
Phoenicolacerta kulzeri FJ460596
Podarcis muralis NC 011607
Podarcis siculus FJ460598
Eremias dzungarica MW250881
Eremias yarkandensis OK585048
Eremias przewalskii KM507330
Eremias multiocellata KJ664798
Eremias multiocellata MK261077
Eremias stummeri KT372881
Eremias szczerbaki OL457296
Eremias nikolskii OK587334
Eremias argus JQ086345
Eremias brenchleyi EF490071
Eremias arguta KU605241
Eremias grammica KU585904
Eremias scripta
Eremias velox KM359148
Eremias vermiculata KM104865
Acanthodactylus boskianus MW496112
Acanthodactylus schmidti MW496124
Acanthodactylus aureus MW496121
Acanthodactylus erythrurus MW496113
Acanthodactylus guineensis MW496123
Mesalina olivieri MW496114
Australolacerta australis MW496118
Meroles squamulosus MW496120
Pedioplanis laticeps MW496119
Gallotia atlantica MW496111
Psammodromus algirus MW496117
Lacertinae
Eremiainae
Gallotiinae
0.1 substitutions/site
