# Supplementary material for: Complete Mitochondrial Genomes of Five Racerunners (Lacertidae: Eremias) and Comparison with Other Lacertids: Insights into the Structure and Evolution of the Control Region
Source: Genes (Basel). 2022 Apr 21;13(5):726. doi: 10.3390/genes13050726 (PMC9141765; doi:10.3390/genes13050726)
Supplement: Supplementary file 1 [file genes-13-00726-s001.zip › genes-1679660-Table S1.pdf]

Table S1. Best-fit models and partitioning schemes selected by PartitionFinder 2.

(a) for BI

Partition No.	Partition identity	Subset	Length (bp)	Best Model
P1	*ATP6*	1-681	681	GTR+I+G
P2	*ATP8*	682-846	165	GTR+I+G
P3	*COI*	847-2403	1557	GTR+I+G
P4	*COII*	2404-3090	687	GTR+I+G
P5	*COIII*	3091-3882	792	GTR+I+G
P6	*CYTB*	3883-5022	1140	GTR+I+G
P7	*ND1, ND4L, ND3, ND5*	5023-5991,7375-7668 7030-7374,9055-10881	3435	GTR+I+G
P8	*ND2*	5992-7029	1038	GTR+I+G
P9	*ND4*	7669-9054	1386	GTR+I+G
P10	*ND6*	10882-11409	528	GTR+I+G

(b) for ML

Partition No.	Partition identity	Subset	Length (bp)	Best Model
P1	*ATP6*	1-681	681	TVM+I+G
P2	*ATP8*	682-846	165	GTR+I+G
P3	*COI*	847-2403	1557	GTR+I+G
P4	*COII*	2404-3090	687	GTR+I+G
P5	*COIII*	3091-3882	792	GTR+I+G
P6	*CYTB*	3883-5022	1140	GTR+I+G
P7	*ND1, ND4L, ND3, ND5*	5023-5991,7375-7668 7030-7374,9055-10881	3435	GTR+I+G
P8	*ND2*	5992-7029	1038	GTR+I+G
P9	*ND4*	7669-9054	1386	GTR+I+G
P10	*ND6*	10882-11409	528	GTR+I+G
