# Supplementary material for: Complete Mitochondrial Genomes of Five Racerunners (Lacertidae: Eremias) and Comparison with Other Lacertids: Insights into the Structure and Evolution of the Control Region
Source: Genes (Basel). 2022 Apr 21;13(5):726. doi: 10.3390/genes13050726 (PMC9141765; doi:10.3390/genes13050726)
Supplement: Supplementary file 1 [file genes-13-00726-s001.zip › genes-1679660-Table S2.pdf]

Table S2. Organization of five mitochondrial genomes of *Eremias* determined in this study.

(a) *E. scripta* KZL15

Gene/Element	Position		Length(bp)	Codon		Intergenic nucleotide	Anticodon	Strand
	From	To		Start	Stop			
tRNA-Phe	1	73	73			-1	GAA	H
12S rRNA	73	1023	951			-4		H
tRNA-Val	1020	1086	67			-5	TAC	H
16S rRNA	1082	2623	1542			-1		H
tRNA-Leu2	2623	2695	73			0	TAA	H
ND1	2696	3664	969	ATG	TAA	+5		H
tRNA-Ile	3670	3741	72			+8	GAT	H
tRNA-Gln	3750	3820	71			-1	TTG	L
tRNA-Met	3820	3887	68			0	CAT	H
ND2	3888	4920	1033	ATG	T--	0		H
tRNA-Trp	4921	4987	67			+1	TCA	H
tRNA-Ala	4989	5057	69			+2	TGC	L
tRNA-Asn	5060	5130	71			+1	GTT	L
rep-origin	5132	5159	28			0		-
tRNA-Cys	5160	5221	62			+4	GCA	L
tRNA-Tyr	5226	5290	65			+1	GTA	L
COI	5292	6836	1545	GTG	AGG	-5		H
tRNA-Ser2	6832	6901	70			+3	TGA	L
tRNA-Asp	6905	6976	72			0	GTC	H
COII	6977	7664	688	ATG	T--	0		H
tRNA-Lys	7665	7731	67			+1	TTT	H
ATP8	7733	7894	162	ATG	TAA	-10		H
ATP6	7885	8565	681	ATG	TAA	-1		H
COIII	8565	9348	784	ATG	T--	0		H
tRNA-Gly	9349	9417	69			0	TCC	H
ND3	9418	9763	346	ATG	T--	0		H
tRNA-Arg	9764	9829	66			+1	TCG	H
ND4L	9831	10127	297	ATG	TAA	-7		H
ND4	10121	11501	1381	ATG	T--	0		H
tRNA-His	11502	11570	69			0	GTG	H
tRNA-Ser1	11571	11636	66			-1	GCT	H
tRNA-Leu1	11636	11706	71			+3	TAG	H
ND5	11710	13533	1824	ATG	TAA	-5		H
ND6	13529	14050	522	ATG	AGG	0		L
tRNA-Glu	14051	14117	67			+1	TTC	L
CYTB	14119	15261	1143	ATG	TAG	-1		H
tRNA-Thr	15261	15331	71			0	TGT	H
tRNA–Pro	15332	15400	69			0	TGG	L
CR	15401	19824	4424			-		-

(b) *E. scripta* KZL44

Gene/Element	From	To	Length(bp)	Codon		Intergenic nucleotide	Anticodon	Strand
				Start	Stop			
tRNA-Phe	1	73	73			-1	GAA	H
12S rRNA	73	1023	951			-4		H
tRNA-Val	1020	1086	67			-5	TAC	H
16S rRNA	1082	2623	1542			-1		H
tRNA-Leu2	2623	2695	73			0	TAA	H
ND1	2696	3664	969	ATG	TAA	+5		H
tRNA-Ile	3670	3741	72			+8	GAT	H
tRNA-Gln	3750	3820	71			-1	TTG	L
tRNA-Met	3820	3887	68			0	CAT	H
ND2	3888	4920	1033	ATG	T--	0		H
tRNA-Trp	4921	4987	67			+1	TCA	H
tRNA-Ala	4989	5057	69			+2	TGC	L
tRNA-Asn	5060	5130	71			-1	GTT	L
rep-origin	5130	5159	30			0		-
tRNA-Cys	5160	5221	62			+4	GCA	L
tRNA-Tyr	5226	5290	65			+1	GTA	L
COI	5292	6836	1545	GTG	AGG	-5		H
tRNA-Ser2	6832	6901	70			+3	TGA	L
tRNA-Asp	6905	6976	72			0	GTC	H
COII	6977	7664	688	ATG	T--	0		H
tRNA-Lys	7665	7731	67			+1	TTT	H
ATP8	7733	7894	162	ATG	TAA	-10		H
ATP6	7885	8565	681	ATG	TAA	-1		H
COIII	8565	9348	784	ATG	T--	0		H
tRNA-Gly	9349	9417	69			0	TCC	H
ND3	9418	9763	346	ATG	T--	0		H
tRNA-Arg	9764	9829	66			+1	TCG	H
ND4L	9831	10127	297	ATG	TAA	-7		H
ND4	10121	11501	1381	ATG	T--	0		H
tRNA-His	11502	11570	69			0	GTG	H
tRNA-Ser1	11571	11636	66			-1	GCT	H
tRNA-Leu1	11636	11706	71			+3	TAG	H
ND5	11710	13533	1824	ATG	TAA	-5		H
ND6	13529	14050	522	ATG	AGG	0		L
tRNA-Glu	14051	14117	67			+1	TTC	L
CYTB	14119	15260	1142	ATG	TA-	0		H
tRNA-Thr	15261	15331	71			0	TGT	H
tRNA–Pro	15332	15400	69			0	TGG	L
CR	15401	19381	3981			-		-

(c) *E. nikolskii*

Gene/Element	Position		Length	Codon		Intergenic	Anticondon	Strand
	From	To	(bp)	Start	Stop	nucleotide		
tRNA-Phe	1	72	72			0	GAA	H
12S rRNA	73	1023	951			0		H
tRNA-Val	1024	1089	66			0	TAC	H
16S rRNA	1090	2634	1545			0		H
tRNA-Leu2	2635	2707	73			0	TAA	H
ND1	2708	3676	969	ATG	TAA	+6		H
tRNA-Ile	3683	3754	72			-1	GAT	H
tRNA-Gln	3754	3824	71			-1	TTG	L
tRNA-Met	3824	3891	68			0	CAT	H
ND2	3892	4924	1033	ATG	T--	0		H
tRNA-Trp	4925	4994	70			+1	TCA	H
tRNA-Ala	4996	5064	69			+1	TGC	L
tRNA-Asn	5065	5137	73			0	GTT	L
rep-origin	5138	5164	70			0		-
tRNA-Cys	5165	5226	62			+5	GCA	L
tRNA-Tyr	5232	5295	64			+1	GTA	L
COI	5297	6841	1545	GTG	AGG	-5		H
tRNA-Ser2	6837	6906	70			+3	TGA	L
tRNA-Asp	6910	6982	73			0	GTC	H
COII	6983	7670	688	ATG	T--	0		H
tRNA-Lys	7671	7735	65			+1	TTT	H
ATP8	7737	7898	162	ATG	TAA	-10		H
ATP6	7889	8569	681	ATG	TAA	-1		H
COIII	8569	9352	784	ATG	T--	0		H
tRNA-Gly	9353	9421	69			0	TCC	H
ND3	9422	9767	346	ATG	T--	0		H
tRNA-Arg	9768	9834	67			+1	TCG	H
ND4L	9836	10132	297	ATG	TAA	-7		H
ND4	10126	11506	1381	ATG	T--	0		H
tRNA-His	11507	11575	69			0	GTG	H
tRNA-Ser1	11576	11641	66			-1	GCT	H
tRNA-Leu1	11641	11711	71			+3	TAG	H
ND5	11715	13538	1824	ATG	TAA	-5		H
ND6	13534	14055	522	ATG	AGG	0		L
tRNA-Glu	14056	14124	69			0	TTC	L
CYTB	14125	15267	1143	ATG	TAG	-1		H
tRNA-Thr	15267	15336	70			0	TGT	H
tRNA–Pro	15337	15404	68			0	TGG	L
CR	15405	20840	5436					-

(d) *E. szczrbaki*

Gene/Element	Position		Length(bp)	Codon		Intergenic nucleotide	Anticodon	Strand
	From	To		Start	Stop			
tRNA-Phe	1	73	73			0	GAA	H
12S rRNA	74	1024	951			0		H
tRNA-Val	1025	1090	66			+1	TAC	H
16S rRNA	1092	2628	1537			0		H
tRNA-Leu2	2629	2701	73			0	TAA	H
ND1	2702	3670	969	ATG	TAA	+4		H
tRNA-Ile	3675	3746	72			0	GAT	H
tRNA-Gln	3747	3817	71			-1	TTG	L
tRNA-Met	3817	3884	68			0	CAT	H
ND2	3885	4917	1033	ATG	T--	0		H
tRNA-Trp	4918	4985	68			+1	TCA	H
tRNA-Ala	4987	5055	69			+2	TGC	L
tRNA-Asn	5058	5128	71			+1	GTT	L
rep-origin	5130	5159	30			0		-
tRNA-Cys	5160	5221	62			+5	GCA	L
tRNA-Tyr	5227	5289	63			+1	GTA	L
COI	5291	6835	1545	GTG	AGG	-5		H
tRNA-Ser2	6831	6900	70			+3	TGA	L
tRNA-Asp	6904	6976	73			0	GTC	H
COII	6977	7664	688	ATG	T--	0		H
tRNA-Lys	7665	7729	65			+1	TTT	H
ATP8	7731	7892	162	ATG	TAA	-10		H
ATP6	7883	8563	681	ATG	TAA	-1		H
COIII	8563	9346	784	ATG	T--	0		H
tRNA-Gly	9347	9415	69			0	TCC	H
ND3	9416	9761	346	ATG	T--	0		H
tRNA-Arg	9762	9826	65			+1	TCG	H
ND4L	9828	10124	297	ATG	TAA	-7		H
ND4	10118	11498	1381	ATG	T--	0		H
tRNA-His	11499	11567	69			0	GTG	H
tRNA-Ser1	11568	11633	66			-1	GCT	H
tRNA-Leu1	11633	11703	71			+3	TAG	H
ND5	11707	13530	1824	ATG	TAA	-5		H
ND6	13526	14047	522	ATG	AGG	0		L
tRNA-Glu	14048	14116	69			+1	TTC	L
CYTB	14118	15260	1143	ATG	TAA	0		H
tRNA-Thr	15261	15330	70			0	TGT	H
tRNA–Pro	15331	15399	68			0	TGG	L
CR	15400	19650	4251					-

(e) *E. yarkandensis*

Gene/Element	Position		Length(bp)	Codon		Intergenic nucleotide	Anticodon	Strand
	From	To		Start	Stop			
tRNA-Phe	1	73	73			0	GAA	H
12S rRNA	74	1024	951			-1		H
tRNA-Val	1024	1090	67			+1	TAC	H
16S rRNA	1092	2634	1543			0		H
tRNA-Leu2	2635	2707	73			0	TAA	H
ND1	2708	3676	969	GTG	TAA	+4		H
tRNA-Ile	3681	3752	72			0	GAT	H
tRNA-Gln	3753	3823	71			-1	TTG	L
tRNA-Met	3823	3890	68			0	CAT	H
ND2	3891	4923	1033	ATG	T--	0		H
tRNA-Trp	4924	4991	68			+1	TCA	H
tRNA-Ala	4993	5061	69			+2	TGC	L
tRNA-Asn	5064	5134	71			+1	GTT	L
rep-origin	5136	5164	29			0		-
tRNA-Cys	5165	5226	62			+5	GCA	L
tRNA-Tyr	5232	5295	64			+1	GTA	L
COI	5297	6841	1545	GTG	AGG	-5		H
tRNA-Ser2	6837	6906	70			+3	TGA	L
tRNA-Asp	6910	6982	73			0	GTC	H
COII	6983	7670	688	ATG	T--	0		H
tRNA-Lys	7671	7735	65			+1	TTT	H
ATP8	7737	7898	162	ATG	TAA	-10		H
ATP6	7889	8569	681	ATG	TAA	-1		H
COIII	8569	9352	784	ATG	T--	0		H
tRNA-Gly	9353	9421	69			0	TCC	H
ND3	9422	9767	346	ATG	T--	0		H
tRNA-Arg	9768	9832	65			+1	TCG	H
ND4L	9834	10130	297	ATG	TAA	-7		H
ND4	10124	11504	1381	ATG	T--	0		H
tRNA-His	11505	11573	69			0	GTG	H
tRNA-Ser1	11574	11639	66			-1	GCT	H
tRNA-Leu1	11639	11709	71			+3	TAG	H
ND5	11713	13536	1824	ATG	TAA	-5		H
ND6	13532	14053	522	ATG	AGG	0		L
tRNA-Glu	14054	14122	69			+1	TTC	L
CYTB	14124	15266	1143	ATG	TAA	0		H
tRNA-Thr	15267	15335	69			0	TGT	H
tRNA–Pro	15336	15404	69			0	TGG	L
CR	15405	18743	3339					-
