# Supplementary material for: Complete Mitochondrial Genomes of Five Racerunners (Lacertidae: Eremias) and Comparison with Other Lacertids: Insights into the Structure and Evolution of the Control Region
Source: Genes (Basel). 2022 Apr 21;13(5):726. doi: 10.3390/genes13050726 (PMC9141765; doi:10.3390/genes13050726)
Supplement: Supplementary file 1 [file genes-13-00726-s001.zip › genes-1679660-Table S3.pdf]

Table S3. The A+T composition of the complete mitogenomes, PCGs, rRNAs, tRNAs and CR, and composition skew values in Lacertidae.

Species	Total			PCGs		rRNAs	tRNAs	CR
	A+T (%)	AT-skew	GC-skew	Number of amino acid	A+T (%)	A+T (%)	A+T (%)	A+T (%)
Acanthodactylus aureus	59.65	0.010	−0.319	11371	59.15	57.79	60.05	68.2
Ac. boskianus	57.95	0.041	−0.352	11369	57.32	55.69	58.99	68.3
Ac. erythrurus	60.90	0.007	−0.313	11373	61.04	58.34	60.21	66.6
Ac. guineensis	60.81	0.025	−0.319	11323	60.79	60.24	60.84	65.9
Ac. schmidti	56.88	0.056	−0.343	11340	55.79	55.87	59.18	66.5
Australolacerta australis	67.85	0.024	−0.335	11367	62.37	59.44	61.76	67.8
Algyroides nigropunctatus	59.78	0.048	−0.341	11355	59.50	58.57	60.18	76.7
Darevskia armeniaca	60.40	0.056	−0.359	11352	60.30	58.64	59.64	66.6
D. brauneri	60.14	0.058	−0.344	11371	59.80	58.46	58.45	67.4
D. caucasica	59.53	0.057	−0.348	11370	60.05	58.55	58.50	57.6
D. chlorogaster	59.14	0.058	−0.338	11370	58.87	58.06	57.47	63.6
D. clarkorum	60.24	0.066	−0.352	11370	60.49	58.70	58.60	64.7
D. daghestanica	60.14	0.063	−0.356	11370	59.97	58.12	58.80	66.5
D. dahli	60.46	0.058	−0.359	11352	60.30	58.68	59.61	65.6
D. derjugini	60.17	0.053	−0.339	11370	59.93	58.54	58.57	66.4
D. mixta	60.43	0.058	−0.359	11352	60.26	58.60	59.55	66.5
D. parvula	59.41	0.077	−0.368	11355	59.21	57.75	58.72	67.1
D. portschinskii	59.58	0.075	−0.373	11367	59.33	58.28	59.57	65.7
D. praticola	58.29	0.067	−0.334	11376	57.87	57.60	57.90	66.1
D. raddei	60.46	0.087	−0.419	11370	59.54	58.52	58.77	64.4
D. rudis	59.83	0.077	−0.372	11370	59.45	57.77	59.48	67.2
D. saxicola	60.19	0.066	−0.352	11355	59.83	58.51	59.01	67.5
D. unisexualis	60.24	0.094	−0.435	11357	59.44	58.25	59.91	66.1
D. valentini	59.82	0.072	−0.367	11370	59.45	58.12	59.33	67.4
Eremias argus	58.38	0.025	−0.328	11387	57.35	55.84	58.23	64.4
E. brenchleyi	58.43	0.031	−0.326	11369	56.84	55.90	59.46	64.3
E. dzungarica	60.04	0.054	−0.341	11375	57.35	56.60	59.05	69.3
E. multiocellata	59.46	0.048	−0.339	11375	57.45	56.94	59.34	66.6
E. nikolskii	58.54	0.043	−0.351	11374	56.08	55.45	58.27	65.3
E. przewalskii	58.19	0.042	−0.334	11375	57.27	56.58	60.16	62.9
E. scripta KZL15	61.33	0.046	−0.324	11375	58.65	55.76	59.71	71.5
E. scripta KZL44	61.16	0.046	−0.325	11374	58.71	56.76	59.74	71.5
E. stummeri	59.62	0.049	−0.347	11374	57.55	56.15	59.27	67.7
E. szczrbaki	59.47	0.053	−0.347	11375	57.00	57.07	58.86	67.8
E. vermiculata	59.82	0.034	−0.355	11375	58.29	56.50	58.52	66.0
E. yarkandensis	59.46	0.046	−0.343	11375	57.55	57.26	58.96	68.0
Gallotia atlantica	57.61	0.135	−0.356	11379	57.89	57.31	56.69	
Lacerta agilis	60.31	0.038	−0.330	11368	59.98	58.70	59.38	65.9
L. bilineata	59.54	0.050	−0.349	11369	59.06	58.46	58.42	70.2
L. viridis viridis	59.95	0.048	−0.341	11355	59.44	57.82	58.82	66.1
Meroles squamulosus	59.35	0.047	−0.356	11366	59.23	57.54	60.40	64.6
Mesalina olivieri	60.04	0.040	−0.336	11376	59.92	58.06	60.04	66.0
Pedioplanis laticeps	59.78	0.052	−0.344	11369	59.75	57.73	60.11	64.3
Phoenicolacerta kulzeri	60.47	0.035	−0.325	11366	60.36	59.09	60.44	63.5
Podarcis muralis	61.45	0.033	−0.333	11367	62.18	59.17	59.23	61.6
Po. siculus	60.80	0.040	−0.348	11370	60.94	59.59	59.79	62.7
Psammodromus algirus	60.70	0.118	−0.345	11375	60.71	59.31	59.79	68.3
Takydromus amurensis	60.03	0.040	−0.304	11375	59.62	58.24	59.87	65.5
T. kuehnei	61.34	0.005	−0.284	11369	61.18	59.06	61.24	66.0
T. septentrionalis	61.87	0.021	−0.307	11372	61.69	60.11	60.95	65.0
T. sexlineatus	62.28	0.011	−0.308	11365	62.85	60.43	62.07	57.9
T. sylvaticus	60.26	0.032	−0.310	11370	60.17	58.94	59.81	62.5
T. tachydromoides	60.34	0.023	−0.296	11374	59.29	59.47	59.75	66.5
T. wolteri	61.99	0.010	−0.290	11372	62.22	59.70	61.10	64.0
Zootoca vivipara	63.24	0.040	−0.320	11375	63.62	60.50	60.66	68.1
