# Supplementary material for: Complete Mitochondrial Genomes of Five Racerunners (Lacertidae: Eremias) and Comparison with Other Lacertids: Insights into the Structure and Evolution of the Control Region
Source: Genes (Basel). 2022 Apr 21;13(5):726. doi: 10.3390/genes13050726 (PMC9141765; doi:10.3390/genes13050726)
Supplement: Supplementary file 1 [file genes-13-00726-s001.zip › genes-1679660-Table S4.pdf]

Table S4. Position of conserved structural elements of the CR in Lacertidae.

Species	TAS 1		TAS 2		CSB 1		CSB 2		CSB 3	
	**From**	**To**	**From**	**To**	**From**	**To**	**From**	**To**	**From**	**To**
Acanthodactylus aureus	15	38	83	92	726	763	1012	1029	1065	1084
Ac. boskianus	15	38	83	92	725	762	1013	1030	1066	1085
Ac. erythrurus	15	38	83	92			252	269		
Ac. guineensis	15	38	64	73	725	763				
Ac. schmidti	15	38	83	92	724	761	1010	1027	1063	1082
Australolacerta australis	15	38	108	117			1011	1028	1065	1084
Algyroides nigropunctatus	15	38	116	125						
Darevskia armeniaca	264	287	357	366	979	1016	1266	1283	1320	1339
D. brauneri	119	142	212	221	834	871	1121	1138	1175	1194
D. caucasica			257	266	691	728				
D. chlorogaster	629	652	1585	1594	1342	1379	1629	1646	1683	1702
D.clarkorum			521	530					560	579
D. daghestanica	325	348	419	428	1039	1076	1326	1343	1380	1399
D. dahli	262	285	355	364	977	1014	1264	1281	1318	1337
D. derjugini	175	198	1447	1456	812	849	1099	1116	1153	1172
D. mixta	266	289	359	368	981	1018	1268	1285	1322	1341
D. parvula	258	281	351	360	973	1010	1260	1277	1314	1333
D. portschinskii	278	301	371	380	993	1030	1280	1297	1334	1353
D. praticola			109	118	379	416	665	682	719	738
D. raddei	2935	2958	4285	4294	3649	3686	3936	3953	3990	4009
D. rudis	260	283	353	362	974	1011	1261	1278	1315	1334
D. saxicola	270	293	363	372	985	1022	1272	1289	1326	1345
D. unisexualis	270	293	414	423	984	1021	1271	1288	1325	1344
D. valentini	235	258	328	337	950	987	1236	1253	1290	1309
Eremias argus	695	718	728	737	1404	1441	1690	1707	1744	1763
E. brenchleyi	1029	1049	1822	1831	1736	1773	2043	2060	2097	2116
E. dzungarica	484	507	577	586	1196	1233	1502	1519	1556	1575
E. multiocellata	531	555	581	590	1064	1101	1370	1387	1424	1443
E. nikolskii	1023	1046	1116	1125	1734	1771	2039	2057	2094	2113
E. przewalskii	618	641	711	720	1329	1366	1635	1652	1689	1708
E. scripta KZL15	809	832	877	886	1520	1557	1826	1843	1880	1899
E. scripta KZL44	676	699	744	753	1387	1424	1693	1710	1747	1766
E. stummeri	704	727	753	762	1415	1452	1720	1737	1774	1793
E. szczrbaki	472	495	565	574	1183	1220	1489	1506	1543	1562
E. vermiculata	1104	1127	1197	1206	1816	1853			2167	2186
E. yarkandensis	469	492	562	571	1181	1218	1487	1504	1541	1560
Lacerta agilis	267	290	360	369	980	1017	1178	1295	1333	1352
L. bilineata	4	27	97	106	715	752	1012	1029	1066	1085
L. viridis viridis	321	344	414	423	1034	1071	1342	1359	1397	1416
Meroles squamulosus					713	750	999	1016	1051	1070
Mesalina olivieri	15	38	142	151	725	762	1034	1051	1089	1108

Pedioplanis laticeps	15	38	108	117	725	762	973	990	1065	1084
Phoenicolacerta kulzeri	400	423	494	503	1114	1151	1401	1418	1455	1474
Podarcis muralis	462	485	511	520	1172	1209	1458	1475	1512	1531
Podarcis siculus	432	455	526	535	1144	1181	1430	1447	1484	1503
Psammodromus algirus	15	38	190	199	727	764	1016	1033	1070	1089
Takydromus amurensis			1486	1495	1243	1280	1531	1548	1586	1605
T. kuehnei	400	423	1775	1784	1113	1150	1400	1417	1805	1824
T. septentrionalis	1502	1525	2876	2885	2218	2255	2505	2522	2559	2578
T. sexlineatus			2031	2040						
T. sylvaticus	1040	1063	2414	2423	1756	1793	2043	2060	2097	2116
T. wolteri	1440	1463	2818	2827	2157	2194	2445	2462	2500	2519
Zootoca vivipara	258	281	351	360	971	1008	1258	1275	1313	1332
