# Supplementary material for: Complete Mitochondrial Genomes of Five Racerunners (Lacertidae: Eremias) and Comparison with Other Lacertids: Insights into the Structure and Evolution of the Control Region
Source: Genes (Basel). 2022 Apr 21;13(5):726. doi: 10.3390/genes13050726 (PMC9141765; doi:10.3390/genes13050726)
Supplement: Supplementary file 1 [file genes-13-00726-s001.zip › genes-1679660-Table S5.pdf]

Table S5. Compositions of conserved structures of the CR in Lacertidae.

Species	TAS1	TAS2	CSB1	CSB2	CSB3
Lacerta dugesii	ACTATTATGTATATAGTGCATTAA	CATACATTAA	CTATATGGTATTATTGTCTTAATGCTTGGTAGACATAT	CAAACCCCCCTACCCCCC	TCGCCAAACCCCTAAAACGA
Acanthodactylus aureus	·················A······	·········T	················GA····················	··················	············A·······
Ac. boskianus	·················A······	A········T	················GA····················	··················	············A·······
Ac. erythrurus	·················A······	T······A·T		T····T··T········T	
Ac. guineensis	·················A······	AG········	·····A·······························A		
Ac. schmidti	·················A······	T········T	·················A··········A·········	··················	············A·······
Australolacerta australis	·················A······	···G······		··················	····················
Algyroides nigropunctatus	························	····T···T·			
Darevskia armeniaca	························	T··G······	···············C·T····················	··················	···T······A·········
D. brauneri	························	T··G······	············G·························	··················	····················
D. caucasica		T··C······	···············C·T····················		
D. chlorogaster	························	T···T·····	···············C·T····················	··················	····················
D. clarkorum		T···T·····			····················
D. daghestanica	························	T··G······	···············C·T····················	··················	····················
D. dahli	························	T··G······	···············C·T····················	··················	····················
D. derjugini	························	T·········	···············C·T····················	··················	····················
D. mixta	························	T··G······	···············C·T····················	··················	····················
D. parvula	························	T··G······	···············C·T····················	··················	····················
D. portschinskii	························	T··G······	···············C·T····················	··················	····················
D. praticola		····AC··G·	············G··C·T····················	··················	····················
D. raddei	························	T·········	···············C·T····················	··················	····················
D. rudis	························	T··G······	···············C·T····················	··············T···	····················
D. saxicola	························	T··G······	············G·························	··················	····················
D. unisexualis	························	T········C	···············C·T····················	··················	····················
D. valentini	························	T··G······	···············C·T····················	··················	····················
Eremias argus	·······C·G······C······C	··C··GA···	·········G······A··G··················	····T·T·T·····T···	C·······TT··CG····C·

E. brenchleyi	· · · · · · · · · · · · · · T · · A · · · · · ·	· · A · · T · · · ·	· · · · · · · · G G C · · · · · · · G · · · · · · · · · · · · · · · · · ·	· · · · · T · · · · · G · · · · · T	· · · · · · · · · · T · G · · · · · A ·
E. dzungarica	· ·	T · · · · · · · · T	· · · · T · · · · · · · · · · · · · · G · · · · · · · · · · · · · · · · · ·	· · · · · · · · · · · · · · · · · ·	· · · · · · · · · · · · · · · · · · · ·
E. multiocellata	· · · · · · · · · · · · · · · · · A · · · · · ·	A · · · · · · · · T	· · · · T · · · · G · · · · · · · · · G · · · · · · · · · · · · · · · · · ·	· · · · · · · · · · · · · · · · · ·	· · · · · · · · · · · · · · · · · · · ·
E. nikolskii	· ·	T · · · · · · · · T	· · · · · · · · · · · · · · · · · · · G · · · · · · · · · · · · · · · · · ·	· · · · T · · · · · · · · · · · · ·	· · · · · · · · T · · · · · · · · · · ·
E. przewalskii	· ·	T · · G · · · · · ·	· · · · T · · · · · · · · · · · · · · G · · · · · · · · · · · · · · · · · ·	· · · · · · · · · · · · · · · · · ·	· · · · · · · · · · · · · · · · · · · ·
E. scripta KZL15	· · · · · · · · · · · · · · · · · A · · · · · ·	· · · · · · · A · T	· · · · · · · · · · · · · · · · · · · G · · · · · · · · · · · · · · · · · ·	· · · · · · · · · · · · · · · · · ·	· · · · · · · · · · · · · · · · · · · ·
E. scripta KZL44	· · · · · · · · · · · · · · · · · A · · · · · ·	· · · · · · · A · T	· · · · · · · · · · · · · · · · · · · G · · · · · · · · · · · · · · · · · ·	· · · · · · · · · · · · · · · · · ·	· · · · · · · · · · · · · · · · · · · ·
E. stummeri	· · · · · · · · · · · · · · · · · A · · · · · ·	A · · · · · · · · T	· · · · T · · · · · · · · C · T C A ·	· · · · · · · · · · · · · · · · · ·	· · · · · · · · · · · · · · · · · · · ·
E. szczrbaki	· · · · · · · · · · · · · · C · · A · · · · · ·	T · · · · · · · · T	· · · · T · · · · · · · · · · T A · · G · · · · · · · · · · · · · · · · · ·	· · · · · · · · · · · · · · · · · ·	· · · · · · · · · · · · · · · · · · · ·
E. vermiculata	· · · · · · · · · · · · · · · · · A · · · · · ·	A · · · · · · · · ·	· · · · · · · · · G · · · · · · C · · G · · · · · · · · · · · · · · · · · ·		· · · · · · · · T · · · · · G · · · A G
E. yarkandensis	· · · · · · · · · · · · · · · · · A · · · · · ·	T · · · · · · · · ·	· · · · T · · · · · · · · · · · · · · G · · · · · · · · · · · · · · · · · ·	· · · · · · · · · · · · · · · · · ·	· · · · · · · · · · · · · · · · · · · ·
Lacerta agilis	· ·	T · · G · · · · · ·	· ·	· · · · · · · · · · · · · · · · · ·	· · · · · · · · · · · · · · · · · · · ·
L. bilineata	· ·	T · · · · · · · · ·	· ·	· · · · · · · · · · · · · · · · · ·	· · · · · · · · · · · · · · · · · · · ·
L. viridis viridis	· ·	· · · · · · · · · ·	· ·	· · · · · · · · · · · · · · · · · ·	· · · · · · · · · · · · · · · · · · · ·
Meroles squamulosus			· · · · · · · · · · · · · · · T C · · G · · · · · · · · · · · · · · · · · ·	· · · · · · · · · · · · · · · · · ·	· · · · · · · · · · · · A · · · · · · ·
Mesalina olivieri	· · A T · · · · · · · · · A T · · A · · · · · ·	· · · G T · · · · ·	· · · · · · · · · · · · · · · T C T · G · · · · · · · · C · · · · · · · · ·	· · · · · · · · · · · · · · · · · ·	· · · · · · · · · · · · · · · · · · · ·
Pedioplanis laticeps	· · A · · · · · · · · · · · T · · · · · · · C A	· · · G · · · · · ·	· · · · · · · · · · · · · · · T C · · G · · · · · · · · · · · · · · · · · ·	· · · · · · · · · · · · · · · · · ·	· · · · · · · · · · · · A · · · · · · ·
Phoenicolacerta kulzeri	· ·	T · · · · · · · · ·	· ·	· · · · · · · · · · · · · · · · · ·	· · · · · · · · · · · · · · · · · · · ·
Podarcis muralis	· ·	T · · · · · · · · T	· ·	· · · · · · · · · · · · · · · · · ·	· · · · · · · · · · · · · · · · · · · ·
Podarcis siculus	· ·	T · · G · · · · · ·	· · · · · · · · · · · · · · · T C ·	· · · · · · · · · · · · · · · · · ·	· · · · · · · · · · · · · · · · · · · ·
Psammodromus algirus	· · A T · · · · · · · · · · · · · · · · · · · G	· · · · · · · · C ·	· ·	· · · · · · · · · · · · · · · · · ·	
Takydromus amurensis		T · · · A · · · · ·	· ·	· · · · · · · · · · · · · · · · · ·	· · · · · · · · · · · · · · · · · · · ·
T. kuehnei	· ·	· · · · · · A · · ·	· ·	· · · · · · · · · · · · · · · · · ·	· · · · · · · · · · · · · · · · · · · ·
T. septentrionalis	· ·	· · · · T · · · · ·	· ·	· · · · · · · · · · · · · · · · · ·	· · · · · · · · · · · · · · · · · · · ·
T. sexlineatus		· · · · · T · · · ·			
T. sylvaticus	· ·	· · · · T · · T · ·	· ·	· · · · · · · · · · · · · · · · · ·	· · · · · · · · · · · · · · · · · · · ·
T. wolteri	· ·	· · · · T · · · · ·	· ·	· · · · · · · · · · · · · · · · · ·	· · · · · · · · · · · · · · · · · · · ·
Zootoca vivipara	· · · · · C · · · · · · · · · · · · · · · · · ·	· G · · · · · · · ·	· ·	· · · · · · · · · · · · · · · · · ·	· · · · · · · · · · · · · · · · · · · ·
