# Supplementary material for: Complete Mitochondrial Genomes of Five Racerunners (Lacertidae: Eremias) and Comparison with Other Lacertids: Insights into the Structure and Evolution of the Control Region
Source: Genes (Basel). 2022 Apr 21;13(5):726. doi: 10.3390/genes13050726 (PMC9141765; doi:10.3390/genes13050726)
Supplement: Supplementary file 1 [file genes-13-00726-s001.zip › genes-1679660-Table S6.pdf]

Table S6. Tandem repeats of the CR in species in Lacertidae.

Species	Copy number	Length	Tandem repeat motif
Acanthodactylus boskianus	2.4	11	TTTTCAACAAA
Ac. erythrurus	2.0	56	TATGTATATTGTACATTAATTTATTTACCCCATGAATATCATCTATATACCTACTT
Australolacerta australis	2.8	12	CACTTTTTTGGC
Darevskia armeniaca	2.2	87	AGCACCTACAACCCAGCCGCTAAATGCGGCTTTTTTGCCCAACACACAACAAACAACTCAACTTTAACCTTCCATCCCCTACTTCAC
	2.3	22	CAAAACTTTTAACAAATTTTAC
D. brauneri	3.7	10	AAATTTTAAC
D. caucasica	2.0	63	TACCCAGCCGCTAAATGCGGCTTTTTTGCCTCCAAACACCCAACTAACAACTCAACTTTAACC
	2.3	22	CAAAAATTTTAACAAATTTTAC
D. chlorogaster	1.8	92	TACCCAGCCGCTTAGTGCGGCTTTTTTGCCTCCAAACAACCAACAAACAAGTCAACTTTAACCTATTAACCCCTGCCTTACATGGCACCTTC
	2.6	90	CTCCAAACAACCAACAAACAAGTCAACTTTAACCTGTTAACCCCTGCCACATGGCACCTTCTACCCAGCCGCTTAGTGCGGCTTTTTTGC
D. clarkorum	2.3	22	CAAAACTTTTAAAAATTTTTAC
	2.1	20	ACAAAAAAAACAAAAAATTTA
D. daghestanica	2.0	53	CAACTCAACTTAAACCTCCCAACCCCTACCTTACATTAAACCTCTTACCCAGCCGCTAAACGCGGCTTTTTTGCCTCCAAACATCCAACAAGCAA CTCAACTTAAACCTCCCAACCCCTAC
	2.4	22	CAAAACTTTTAACAAATTTTAC
D. dahli	2.3	22	CAAAACTTTTAACAAATTTTAC
D. derjugini	1.9	90	TTACCTAGCCGCTAAATGCGGCTTTTTTGCCTCCAAACATCCAACAAATAACACAACTTTAACCTTCTAACCCCTGCTTCACTGCACTGG
D. mixta	2.0	87	AACCCAGCCGCTAAATGCGGCTTTTTTGCCCAACACACAACAAACAACTCAACTTTAACCTTCCAACCCCTACTTCACAGCACCTGT
	2.3	22	CAAAACTTTTAACAAATTTTAC
D. parvula	2.6	23	CAAAACTTTTAACAAATTTATAC
D. portschinskii	3.0	23	CAAAACTTTTAACAAATTTTTAC
D. raddei	2.8	59	CACACATTCAACAAATAATTCAACTTTAACCTCCCAATACCTACTACACAAACTGCCAT
	4.8	58	CACACATTCACAAACTATTCAACTTTAACCTCCCAATACCTACAACACAAACTGCCAT
	3.9	59	TGCCTCCACACATTCAACAAATAATTCAACTTTAACCTCCCAATACCTACGACACAAAC
	26.6	58	CAACTTTAACCTCCCAATACCTACAACACAAACTGCCATCACACATTCAACAAACATT
	4.5	61	AACTTTAACCTCCCAATACCTACGACACAAACACTTTGCCATCACATTCAACAAACCATTC
D. rudis	3.0	23	CAAAACTTTTAACAAATTTTTAC
D. saxicola	3.0	22	ACAAAACTTTTAACAAATTTTA
D. unisexualis	2.0	15	AACACCTACTACACC
	2.4	22	CAAAACTTTTAACAAATTTTAC
Eremias argus	2.3	20	AAAAAAATTTTTTTATAATA
	7.8	65	CCTAAAACCAGGCTGCCCAAGCAGCCGGATTCTTACTCCCCAAGCCGCTTCTAGCGGCTTTTTTG

E. brenchleyi	65	13.5	AAAACTAGCCGCCCCAGCGGCCTAATTCTTCCTACCCCAGCCGCTTCTAGCGGCTTTTTTGCCTT
	19	2.1	TACATAATACATAACTGTT
	12	2.5	TTTGTTAAAACC
	56	14.3	ACAAAGATCAATTCGTTTGTTGTTAACCTAAATTTCTCTAGAACACAAACAAATTG
	19	2.8	TTTTTAATTAAAAATATTT
	10	5.1	AATTTTTTTT
	19	1.9	AAAATAATTTTTAATTTTT
E. dzungarica	57	6.5	CCTAAAAACAGGCCGCTCAGGCGGCCCACTACCCAGCCGCTTATAGCGGCTTTTTTG
	56	17.0	TTAACAAAGAACAATTCGTTTGTTATTGTCCTAAACTTATCTAGAACACAAATAAA
	20	2.4 ×2	AAAAAATTTCTTTATTTTCA
E. multiocellata	61	6.7	CCTAAAAACAGGCCGCCCCAGCGGCCCATTACTCCCCCTGCCGCTTATAGCGGCTTTTTTG
	56	13.8	AACAAAGAACAATTCGTTTGTTGTTAACCTAAACTTCTCTAGAACACAAACGAATT
E. nikolskii	65	12.6	CCCTAAAACAGGCCGCTCCAGCGGCTTACCCCACCCTTCACCAGCCGCTTCTAGCGGCTTTTTTG
	56	8.9	AACAAATTAACAAAGAACAACTTGTTTGTTATTAACCTAAACTTCTCGGGAACACA
	20	2.5 ×3	TTTAAAAAAAATTTTTCTATT
E. przewalskii	66	7.8	CCTAAAAACAGGCCGCTCCGACGGCCCCTTGCCCCCTCCACCCCGCCGCTTATAGCGGCTTTTTTG
	56	12.0	ACAAAGAACAATTCGTTTGTTATTGTCCTAAACTTATCTAGAACACAAACGAATT
	18	1.9	TAAAAATAATTTTTTTAT
E. scripta KZL15	67	10.5	CCTTTAAAACAAGCCGTCCAAGACGGCATGCATTCCCCTTCACCGGCCGCTTCTAGCGGCTTTTTTG
	11	4.2	TTTTTAACAAA
	155	13.7	AAAGGTTAATTTGTTTATAATTAACCTAAATTTGTCTAGAACACAAACAAATTAATAAAAGAACTAATTTGTTTATAATTGACTTAAATTTACCTAGAACACAAACAAATTAACAAAGATTAATTCATTTGTAACTAACTAAATTTTAAATTTTA
E. scripta KZL44	67	8.6	CCTTTAAAACAAGCCGTCCAAGACGGCATGCATTCCCCTTCACCGGCCGCTTCTAGCGGCTTTTTTG
	11	4.2	TTTTTAACAAA
	155	11.7	AAAGGTTAATTTGTTTATAATTAACCTAAATTTGTCTAGAACACAAACAAATTAATAAAAGAACTAATTTGTTTATAATTGACTTAAATTTACCTAGAACACAAACAAATTAACAAAGATTAATTCATTTGTAACTAACTAAATTTTAAATTTTA
E. stummeri	54	10.3	CCTACAACCAACCATCCCCATACATTTCACAAGCCGCTTCTAGCGGCTTTTTTG
	56	22.1	TCTCTAGAACACAAACAAACTAACAAAGAACAATTCGTTTGTTGTTAACCTAGATT
	11	2.9	TTTTTTAAATA
E. szczrbaki	61	5.9	CCTTAAAACCAGCCGCCCAACGGCCCTATTTCACTCCAAGCCGCGCATAGCGGCTTTTTTG
	56	12.2	ACAAACGAATTCACAAAGAACAATTCGTTTGTTATTAACCTAAACTTCTCTAGAAC
	21	2.1 ×2	AAAAATTTTTATTTTTTAAAA
E. vermiculata	55	18.3	TCCACTTCTCACCCTATTTCCTCTACTTCACATGCCGCTCCTAGCGGCTTTTTTG
	56	13.1	TCTAGAACACAAACGAATTAACAAAGAACAATTCGTTTGTAATTAACCTAAGTTTC
	56	6.1	TTCTCTAGAACACAAACGAATTAACAAAGAACAATTCGTTTGTAATTAACCTAGGT
	10	4.1	TAAATATTTT
E. yarkandensis	65	5.8	CCTAAAAACAGGCCGCTCCGGCGGCCCATTGCCCCCTTCCCCAGCCGCTTCTAGCGGCTTTTTTG
	56	11.3	TTAACAAACAACAATTCGTTTGTTATTATCCTAAACTTATCTAGAACACAAATAAA
	19	2.4	CAAAAAAAATTTCTTATTTT

Lacerta agilis	36	6.5	ACCCAGCCGCTCCAAGAGCGGCTTTTTTGCCTCTAC
	11	3.4	TTTTAACAAAC
L. bilineata	11	4.1	ACAAATTTTTA
L. viridis viridis	11	4.1	ACAAATTTTTA
Pedioplanis laticeps	13	3.1	TAAAAAATTTTTT
Phoenicolacerta kulzeri	34	8.7	AGCGGCTTTTTACCTCCCCCCACCAGCCACTCTT
Podarcis muralis	34	11.7	CCACCTGCCGCTCCCAGCGGCTTTTTTGCCTCCA
	11	2.5	AAATTTTAACA
Podarcis siculus	36	11.4	CCAGCCGCTCCACAAGCGGCTTTTTTGCCTCCCTAC
Psammodromus algirus	14	2.1	AAATAAAAAACAAC
Takydromus amurensis	67	3.6	TAACACATGCCGCTACAAGCGGCCTATTTTCCTCTACCATTAACAAGTAAATACAAACATATATCTC
	80	2.3	ATATATGTTTAATCGTGCATTAACTTATTTACCCCATGAAAAATATATATATACAGTATCCTAATAATAAGACATAATAC
	20	2.0	AATTTTGAAAAATTTCGTCC
T. kuehnei	70	2.2	ACTTTTGTATGCCGCCTTACGCGGCTTATTTTCCCCAGCTATTAGTTTGTAATTGATCAATTGGTTTGTA
	70	2.0	TATTAGTCTGTAATTGATCAATTGGTTTGTAACTTTTGTATGCCGCCTTGCGCGGCTTATTTTCCCCTGC
T. septentrionalis	68	18.2	ATTTTCCTCTAGCATTAATCAAACTAATACAAGTACCTATCTACAAACCATGCCGCTATACGCGGCTT
T. sexlineatus	68	30.0	TTTTTACATGCCGCCTCGCGCGGCTTATTTTCCCCATCTCAAATAATGAACAAACAAACATACTTTCC
T. sylvaticus	69	8.1	ATTTTCCTCTACTCCTACACAAGTTAATACACTCCAACCATATTCAACACATGCCGCATCCTGCGGCTT
	69	5.1	TCCTGCGGCTTATTTTCCTCTACTCCTACACAAGTTAATACACTCCAACCATATTCAACACATGCCGCA
T. tachydromoides	65	8.0	TTTTCCTCTACATTAAACAAGTAAATACAAGAATTTTACTATCTCATGCCGCTATAAGCGGCTTA
T. wolteri	67	17.4	TAACCATGCCGCTCTATGCGGCTTTTTTACCTCTACCACTACTTGCACAAATACAAATAATACCCAA
Zootoca vivipara	34	2.6	TTTTGCCTCTACCACTAGCCGCTTTATGCGGCTT

Note:
The motif segment "TAGCGGCTTTTTTG" present in all examined *Eremias* species is in purple.
The motif segment "GCGGCTT" present in seven species of *Takydromus* is in green; segment "TTTTCC" present in six species of *Takydromus* is in blue.
"×2" or "×3"denotes similar type of TR in the species duplicated twice or three times, respectively.
