# Supplementary material for: Complete Mitochondrial Genomes of Five Racerunners (Lacertidae: Eremias) and Comparison with Other Lacertids: Insights into the Structure and Evolution of the Control Region
Source: Genes (Basel). 2022 Apr 21;13(5):726. doi: 10.3390/genes13050726 (PMC9141765; doi:10.3390/genes13050726)
Supplement: Supplementary file 1 [file genes-13-00726-s001.zip › genes-1679660-Table S7.pdf]

Table S7. Uncorrected genetic distances (p-distances) within and between *Pareremias* and *E. nikolskii* based on 13 concatenated PCGs.

	1	2	3	4	5	6	7	8	9	10	11
1. *E. nikolskii*											
2. Viviparous group	0.108										
3. *E. argus-E. brenchleyi*	0.123	0.123									
4. *E. argus*	0.122	–	–	–							
5. *E. brenchleyi*	0.124	–	–	–							
6. *E. szczerbaki*	0.107	–	–	–	–						
7. *E. yarkamdensis*	0.105	–	–	–	–	0.087					
8. *E. dzungarica*	0.106	–	–	–	–	0.088	0.039				
9. *E. prewalskii*	0.109	–	–	–	–	0.091	0.043	0.051			
10. *E. stummeri*	0.109	–	–	–	–	0.067	0.084	0.087	0.089		
11. *E. multiocellata*	0.109	–	–	–	–	0.092	0.079	0.087	0.084	0.091	
